# Umbilical cord blood natural killer cells for adoptive immunotherapy: identifying optimal starting material and processing parameters

**DOI:** 10.3389/fimmu.2025.1734453

**Published:** 2026-01-13

**Authors:** M. G. Kennedy, W. Patterson, S. T. Cox, L. Wynn, C. Stock da Cunha, M. O’Dwyer, R. Danby, D. Hernandez

**Affiliations:** 1 Anthony Nolan Research Institute, Royal Free Hospital, London, United Kingdom; 2 University College London (UCL) Cancer Institute, London, United Kingdom; 3 School of Medicine- University of Galway, Galway, Ireland; 4 ONK Therapeutics, Galway, Ireland; 5 Churchill Hospital, Oxford University Hospitals National Health Service (NHS) Foundation Trust, Oxford, United Kingdom

**Keywords:** adoptive cell therapy, cell donor selection, cryopreservation, natural killer cells, umbilical cord blood

## Abstract

**Introduction:**

Umbilical cord blood (UCB) is an attractive source of natural killer (NK) cells for the development of allogeneic ‘off-the-shelf’ cancer immunotherapies. This is due to the relatively high proportion of highly proliferative NK cells compared to adult peripheral blood (APB), a low risk of graft-versus-host disease and ease of procurement. However, due to the limited starting volume of UCB and naïve phenotype of isolated cells, *ex vivo* NK cell expansion and activation is essential to generate clinically relevant doses of cells with potent anti-tumor activity. Furthermore, intrinsic variability in both *in vitro* and clinical performance of NK cells from different UCB units (CBUs) has been reported.

**Methods:**

To better characterize this variability, we measured UCB NK cell *ex vivo* fold expansion, phenotype and cytotoxic potential using a basic expansion system. We then used these results to identify characteristics related to superior performance, enabling the optimization, selection and processing of CBUs for the manufacture of NK cells as therapies at a larger scale.

**Results:**

Our results revealed that despite wide inter-donor variability in performance between CBUs, *a priori* selection could be used to identify units likely to show high expansion and/or cytotoxicity. We observed that decreased time between UCB collection and CD3^-^ UCB mononuclear cell (CBMC) isolation was associated with significantly higher NK fold expansion (n=13; p<0.05). Furthermore, a cryopreservation step following early isolation and prior to expansion, significantly increased the expansion potential of the isolated NK cells (p<0.05), thus providing an opportunity for pre-selection and parallel culture of multiple optimal units. Finally, the NK cells from CBUs collected from caesarean sections had statistically significantly increased proliferative potential compared to those from vaginal deliveries (n=13; p<0.05).

**Conclusion:**

In conclusion, early isolation and cryopreservation of CD3^-^ CBMCs from caesarean section CBUs offer an optimal starting material for use in UCB-derived NK cell immunotherapies, providing superior *ex vivo* performance and enabling batch testing to selectively expand cells from CBUs with the greatest potential.

## Introduction

Natural Killer (NK) cells are lymphoid cells of the innate immune system and the first line of defense against pathogens, performing immune surveillance and eliminating infected and cancerous cells (1). NK cells constantly sense cells in their environment via a series of germ line encoded activating and inhibitory receptors that enable recognition of self and non self (loss of MHC Class I), with net input of these signals determining their activation status and function. Activated NK cells can lyse and eliminate cells, by engagement of activating receptors on their surface. For example, NKG2D can detect stress ligands such as MICA, MICB, and UL16-binding proteins upregulated by malignant and infected cells. Once activated, NK cells release Perforin and Granzyme leading to target cell lysis and apoptosis, concomitant with the release of IFNγ and TNFα cytokines to recruit other immune cells to the microenvironment (2). Additionally, antibody recognition via the Fc receptor CD16 induces a strong activation signal that can over-ride inhibitory signals. While the majority of the killing is mediated by Granzyme, there is an important contribution from Fas-L and TRAIL via the death receptor pathway (3).

Unlike T cells, NK cells kill in an antigen independent manner and do not require prior sensitization. This is an attractive property in the context of adoptive immunotherapy as they have the potential to be used against multiple types of tumors and can mitigate against immune escape due to either loss of targeted antigen, or downregulation of MHC class I, from the tumor cells which renders them uniquely sensitive to NK cell killing. (2, 4–6). Recently several groups have investigated the use of allogeneic NK cells as adoptive cell immunotherapy against cancers using both adult peripheral blood and umbilical cord blood derived NK cells with promising results (review in (2, 7)).

Umbilical cord blood (UCB) is an attractive source of cells for adoptive immunotherapy as collection poses no risk to the donor and provides a relatively high proportion of NK cells with high proliferative potential and naïve phenotype (8, 9). The key limiting factors of UCB are the low number of cells obtained from a single umbilical cord blood unit (CBU) and intrinsic variability between donors regarding proportion, phenotype and function of these cells (10).

To overcome these limitations, three key strategies can be applied: (a) optimization of isolation and *ex vivo* expansion of NK cells, increasing potential doses produced from a single CBU (11); (b) enhancing cytotoxic potential of cells, increasing their efficacy as a therapy through strategies such as cytokine activation during *ex vivo* expansion, the use of CD16 antibodies or feeder cells (4), as well as incorporation of genetic modifications to enhance NK cell killing capability (11–13); (c)selection of CBUs based on indirect markers of enhanced performance, although currently the factors affecting intrinsic CBU variability are mostly unknown. Metrics for NK cell performance, such as *ex vivo* fold expansion and *in vitro* cytotoxicity, are key for processing optimization and developing a therapy that can be carried forward to preclinical and clinical trials. We report in this study the systematic measurement of NK cell performance parameters: cell expansion and cytotoxicity, on a sizable group of cord blood units and how they correlated to both donor and upstream processing parameters. We identified that minimizing the time between UCB collection and cell isolation had the most impact in improving cell performance.

**Figure 1:**
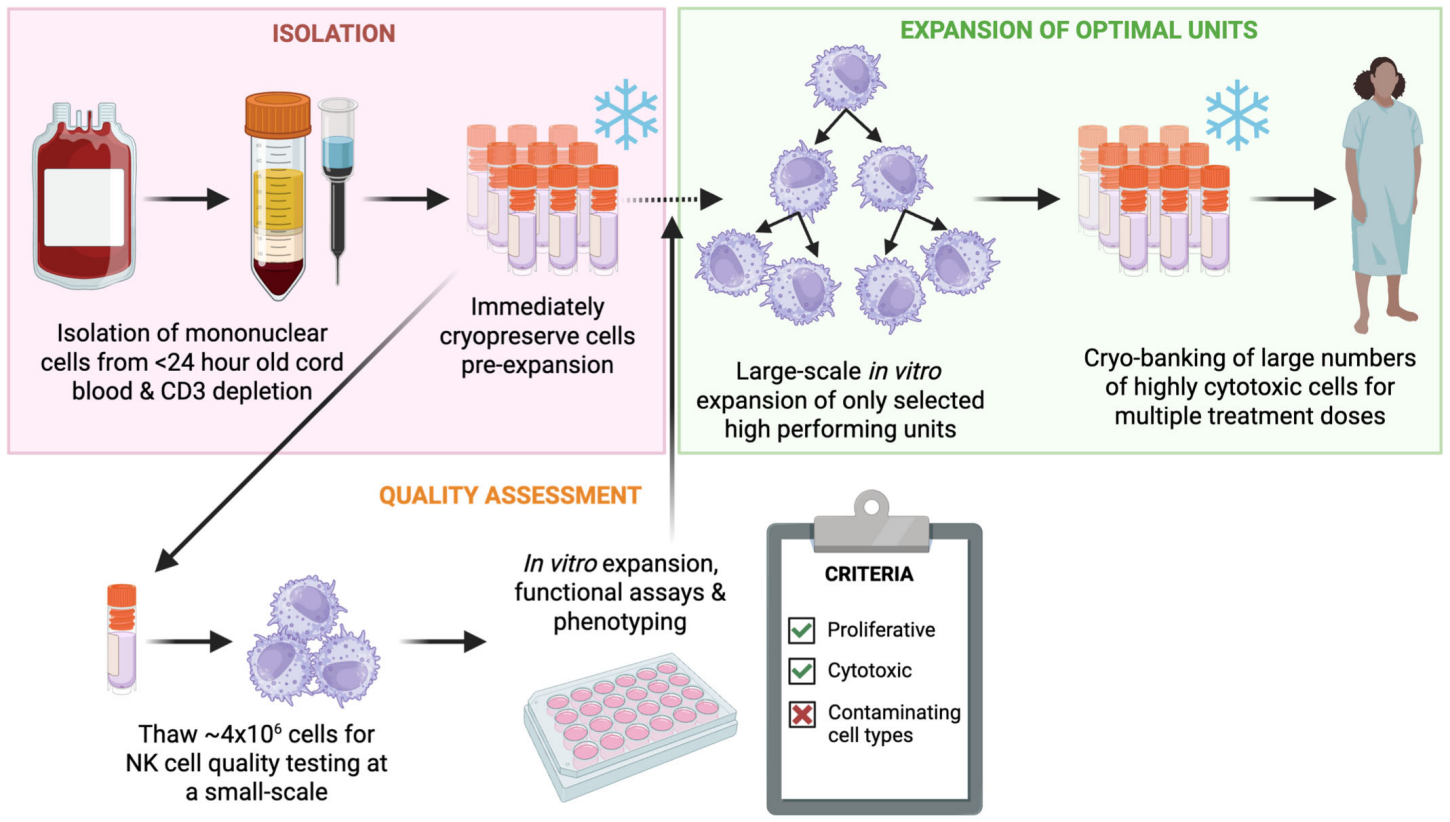
Proposed workflow for selection processing and phased screening of fresh UCBUs as starting material for NK cell therapy manufacturing. The workflow starts with very early (within 24 hours) isolation of CBMCs from fresh UCB units with a CD3 depletion step, samples of the whole blood can at this time also be sent for IDM testing. The isolated CD3- cells, are then cryopreserved in suitable aliquots, with one specifically designed for small scale testing of growth and cytotoxicity capacity. Once a bank has been established, several CBU batches can be tested (small aliquots) and the best ones used for large scale expansion. Expanded batches can then be cryopreserved and used off the shelf for therapy.

We used our findings to delineate a workflow (**Figure 1**) which minimizes the risk of manufacturing failures and subpar performance of UCB NK cells by: selecting fresh CBUs based on a set criterion, isolating CBMCs rapidly following collection (<24h), utilizing a CD3 depletion protocol and introducing a cryopreservation step on the depleted fraction immediately upon isolation. This workflow allows batch testing of small sample aliquots, prior to large scale manufacture, and could ease pressures in the supply chain as cells are cryopreserved. We believe that the suggested workflow (**Figure 1**) would allow for better selection of starting material and for cost savings by only expanding units likely to produce clinically relevant NK cell doses.

## Methods

### Whole blood processing

UCB was collected from healthy donors (n=38) with prior written informed maternal consent, under HTA license 22513 (Anthony Nolan Cord Blood Bank) and with ethical approval of 20/EM/0028 East Midlands Derby NHS Research Ethics Committee UK. CBUs not suitable for banking and cryopreservation, were diverted to processing laboratories, either in the same location or shipped overnight to our research laboratories at a separate location. Upon arrival at the banking facility, fresh CBUs were mixed and 1.5ml was withdrawn from the blood bag using a syringe with Luer lock (OriGen) and sterile connection device (Genesis); the bag was then sealed and transported/stored at 4°C prior to processing. The 1.5ml sample of whole UCB was used to conduct a complete blood count using the XN-1000 hematology analyzer (Sysmex).

Adult peripheral blood (APB) was collected from healthy volunteers (n=5) following prior written informed consent, with ethical approval 19/LO/141 of London Bridge NHS REC, UK. Samples were stored at 4°C prior to processing for 44h.

UCB or APB mononuclear cells (CBMCs and PBMCs, respectively) were isolated from fresh whole blood via centrifugation over a Ficoll-Paque PLUS 1.077g/ml gradient (Cytiva) in Leucosep tubes (Greiner Bio) followed by erythrocyte lysis with Pharmlyse (BD Bioscience).

### T cell depletion of mononuclear cells

Depletion of CD3^+^ cells from CBMCs and PBMCs was undertaken following mononuclear cells isolation. Mononuclear cells were incubated with CD3 antibody-conjugated magnetic microbeads (Miltenyi Biotec) then passed through an LD column in the QuadroMACS separator (Miltenyi Biotec), where the negative fraction was collected. The process was later replicated in both the semi-automated AUTOMACS (Miltenyi Biotech), and the TyTo fluorescent activated cell sorter (FACS; Miltenyi Biotech) using an APC conjugated anti-CD3 antibody (Miltenyi Biotech).

### Cryopreservation of CBMCs

Cells were centrifugated at 400xg for 10 min (room temperature), the supernatant was discarded, then cells were resuspended at 8x10^6^ cells/ml in Cryostor CS10 (STEMCELL Technologies) and aliquoted into cryo vials at 0.5mL/vial. Vials of cells were frozen at -1°C/min to -80°C in a CoolCell (Corning), then transferred to the vapor phase of a liquid nitrogen tank.

### NK cell expansion

Once isolated, CD3^-^ CBMCs were either cultured immediately with no cryopreservation step, or cryopreserved cells were thawed and seeded in culture. Cells that were isolated <24h post UCB collection (19h ±3.3; n=13) are referred to as immediately isolated, whereas cells where CD3^-^ CBMCs was delayed by approximately 2 days (44h ±7.7; n=25) due to delivery between collection center and processing center are referred to as delayed isolations.

CD3^-^ CBMC were seeded at 1x10^6^ cells/ml in NK MACS medium (Miltenyi Biotech) with 10% heat inactivated human AB serum (Sigma-Aldrich), 1% penicillin streptomycin (pen/strep; Corning), supplemented with 1000IU/ml IL-2 and 100ng/ml IL-15 (Peprotech). Cells were cultured for 21 days at 37°C with 5% CO_2_. Culture medium was hemi-depleted every 48–72 hours and fresh medium and cytokines added. These are minimal, non-optimized, conditions for NK cell growth.

CD3^-^ PBMCs were always cultured immediately after isolation (no cryopreservation), in the same conditions as UCB derived cells, except a lower concentration of IL2 was used (500 IU/ml) in accordance with previous studies (14).

### Bioluminescence cytotoxicity assays

Specific target cell lysis of K562 cells was measured at various effector to target (E:T) cell ratios following coculture with NK cells expanded for 7,14 or 21 days, for 3h.

The Toxilight Bioassay kit (Lonza) was used according to manufacturer’s guidelines to calculate the percentage of target specific lysis. Relative bioluminescence was read out on the Varioskan LUX (Thermo Fisher Scientific).

Specific lysis is calculated using the equation below,


$$Specific\;Lysis=100\times \frac{minimum\;death\;RLU-test\;RLU}{minimum\;death\;RLU\;-\;maximum\;death\;RLU}$$

*RLU*= relative light unit measure of bioluminescence

*minimum death RLU* = the bioluminescence of the target only control culture

*test RLU* = bioluminescence of the test co-culture of NK:K562- bioluminescence of NK alone

*maximum death RLU*= bioluminescence of fully lysed control target (K562 cells)

Direct cytotoxicity and ADCC of NK cells against Raji cells was measured using this protocol, except two conditions were tested per CBU. One where Raji target cells were pre-incubated with CD20 antibody Obinutuzumab and a no antibody control.

### Flow-based cytotoxicity assay

Expanded UCB NK cells that were cryopreserved at day 22–24 of expansion were thawed and rested in culture (37°C with 5% CO_2_) for 7 days in NK MACS medium with 10% heat inactivated AB human serum, 1% pen/strep, supplemented with 500IU/ml IL-2 and 100ng/ml IL-15 before enhanced cytotoxicity characterization. Their cytotoxicity against five different target leukemic cell lines was measured: K562, Raji, THP-1, KG-1a and MOLM-13. Target cells were first stained with CellTraceViolet (C34557; Invitrogen) in PBS for 15 min at 37°C, then washed in co-culture medium. As with the bioluminescence assay, NK cells and target cells were seeded at a range of E:Ts alongside target only and effector only controls, then they were incubated for 4h or 24h at 37°C with 5% CO_2_. After the incubation, cells were stained for flow cytometry (gating strategy **Supplementary Figure 1**) to assess target cell viability and NK cell CD107a expression. Target specific lysis was calculated using the following equation (15):


$$Specific\;Lysis=100\;\times \;\frac{no.live\;target\;cells\;(control)-\;no.live\;target\;cells\;(test)}{no.live\;target\;cells\;(control)}$$

### Flow cytometry

UCB and APB cells were washed with FACS buffer (PBS buffer with 3% FBS), then stained for 15 min at 4°C in the dark with one of the antibody cocktails made up in FACS buffer (**Supplementary Table 1**). After staining, cells were washed twice with FACS buffer. Cell viability was assessed with either LIVE/DEAD Fixable Aqua Dead Stain (L34957; Invitrogen) in PBS for 25 min at 4°C, or Zombie NIR Fixable Viability Kit (423106; Biolegend) in PBS for 10 min at room temperature. For intracellular staining panel, cells were fixed at this point with BD Cytofix/Cytoperm kit (554714) according to manufacturer’s instructions, and then stained with antibody cocktail 3a. Lastly, all cells were washed and resuspended in FACS buffer and acquired on the BD Fortessa Flow Cytometer (Cocktails 1 and 2) or the ID7000 Spectral Analyser (Cocktails 3 and 3a, 4 & 5). Data was analyzed using Flowjo version 10.10.00or ID7000 Software (gating strategy in **Supplementary Figure 2**).

### Calculation of NK fold-expansion

To monitor NK cell proliferation during expansion, NK fold expansion was calculated at days 7, 14 and 21 of culture using the following calculation (16):


$$Fold\;Expansion=\;\frac{expanded\;total\;live\;cells}{initial\;total\;live\;cells}\times \frac{final\;proportion\;CD3^{-}CD56^{+}}{initial\;proportion\;CD3^{-}CD56^{+}}$$

### Statistical analysis

All statistical analysis was conducted on Prism 10 version 10.2.3. For all datasets a Shapiro-Wilk test was applied to determine whether data was normally distributed, so that parametric or non-parametric testing could be applied where appropriate. When assessing the variance between two groups two-tailed T tests or Mann Whitney U tests were used, for parametric and non-parametric data, respectively. Whilst for three or more groups, one-way ANOVAs with Tukey’s multiple comparisons tests were applied. Paired or unpaired tests were used for matched and unmatched samples, respectively. Paired statistical tests were used when comparing the n=13 NK cells from immediately isolated starting material (including cryopreserved and non-cryopreserved), whilst unpaired tests were used for comparisons between the cells from delayed isolated n=20something units and the immediately isolated ones. When comparing between different groups at different time points two-way ANOVAs were used with Tukey’s multiple comparisons tests. For correlational analysis to measure the relationship between two variables Pearson or Spearman correlation was used, depending on whether data was parametric or non-parametric. Simple linear regression was conducted to identify potential causal relationships between two variables. Relevant p values and r values are referenced where appropriate in the text, as well as means quoted with ± standard deviation. Significant p-values are denoted with *p<0.05, **p ≤ 0.01, ***p ≤ 0.001 and ****p ≤ 0.0001.

## Results

### Isolation of CD3^-^ cells from fresh UCB units

UCB units not suitable for banking (below TNC threshold for transplant for example) were used to isolate CD3^-^ CBMCs. This process is easily achieved at the banking site, as units arrive. Post Ficoll yields were 1.71x10^8^ ± 6.1x10^7^CBMCS/CBU, and the CD3 depleted fraction yield post isolation was 4.46x10^7^ ± 1.45x10^7^ CD3^-^ CBMCs/1x10^8^ cell input, with a purity (%CD3^-^) of 86.1% ± 9.4 CD3^-^. The proportion of CD3^+^ cell carry over was minimal immediately post isolation when magnetic beads were used (0.91±0.89%), but higher when using FACS (12.2±7.6%). Following cryopreservation, the CD3+ carryover remains at similar levels on D0 (day of thaw) and decreases during the 21 days of culture (**Supplementary Figure 3**).

### CD3^-^ CBMCs provide a prime starting material for the expansion of NK cells

**Figure 2:**
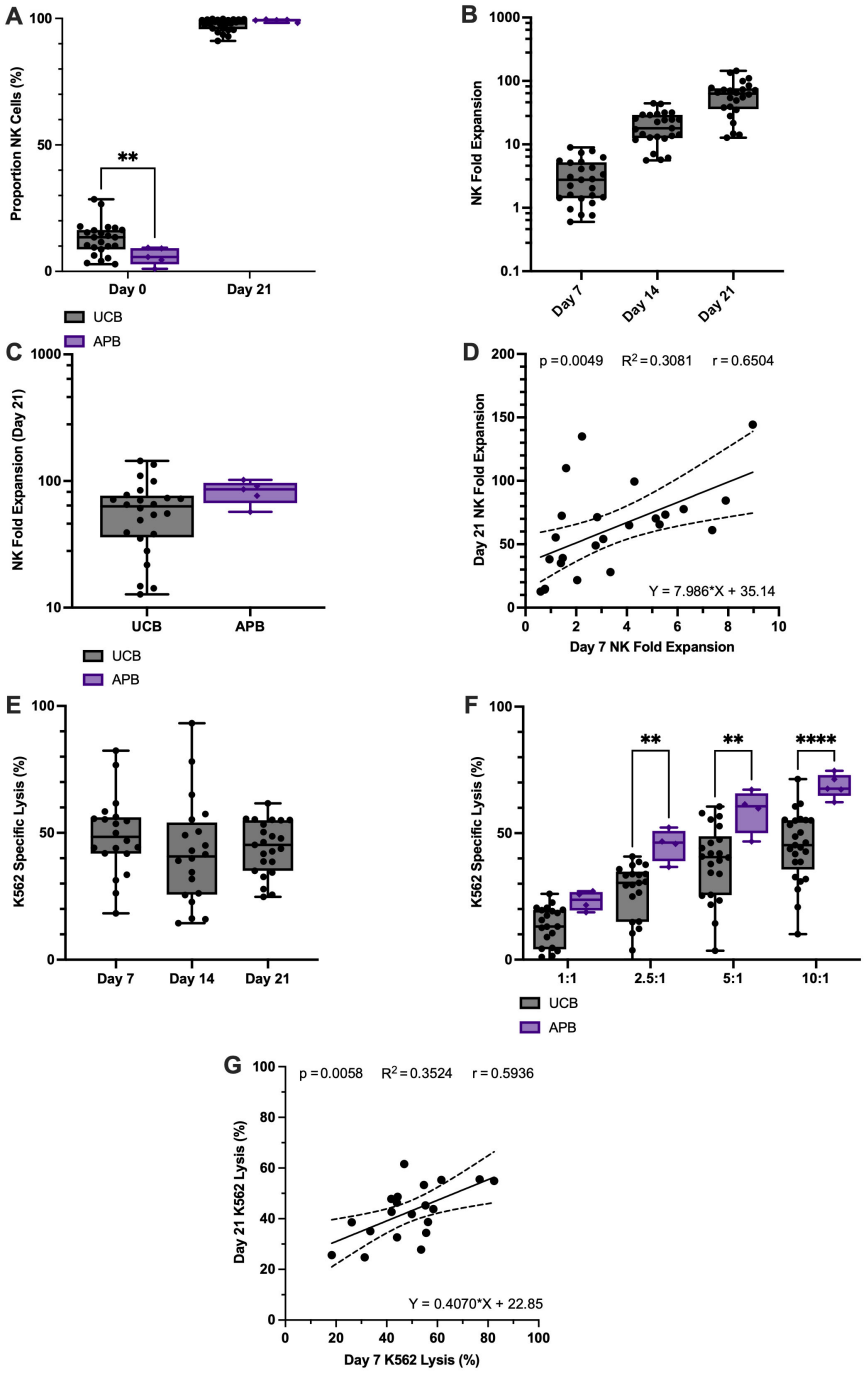
High performance of UCB NK cells in ex vivo fold expansion and cytotoxicity can be predicted early in expansion, despite high inter-donor variability in performance. **(A)** Mononuclear cells isolated from UCB (n=25) or APB (n=5) were CD3+cell depleted and expanded in a feeder free culture system for 21 days until they became >90% CD3-CD56+ NK cells. **(B)** Ex vivo UCB NK cell fold expansion was highly variable between donors throughout the 21-day protocol. **(C)** NK cell fold expansion was comparable between UCB and APB. **(D)** Elevated day 7 NK cell fold expansion was predictive of a high day-21 total NK fold expansion according to correlation and linear regression analysis (solid line). Dashed lines show 95% confidence intervals. **(E)** NK cell cytotoxicity measured via K562 specific lysis in a co-culture assay was variable between different UCB donors throughout the 21-day ex vivo expansion (10:1 target-to-effector ratio; E:T). **(F)** APB derived NK cells demonstrated a higher cytotoxicity at most of the E:Ts tested compared to UCB NK cells. **(G)** Increased K562 specific lysis by CB NK cells at day 7 is predictive of greater day 21 K562 specific lysis according to correlation and linear regression.

Using a basic feeder free expansion system consisting of NK culture media supplemented with IL-2 and IL-15, CD3^-^ CBMCs were cultured for 21 days resulting in an increase in the CD3^-^CD56^+^ NK cell population from 12.8% ± 6.6% at day 0, to 96.8% ± 3.3% at day-21 (**Figure 2A**). As expected, CD3^-^ PBMCs (99.8% ± 0.1% CD3^-^) had a significantly smaller starting proportion of CD3^-^CD56^+^ NK cells, 5.9% ± 3.5% (p<0.01; **Figure 2A**), but they too achieved a high proportion of CD3^-^CD56^+^ NK cells, 98.4% ± 0.2% by day 21. Over the culture period the phenotype of the cell population became more homogeneous, with most cells becoming CD3^-^CD56^+^ NK cells and the total cell number increased considerably. However, substantial inter-donor variability in the fold expansion and the cytotoxic potential of the UCB derived NK cells was identified.

### Inter-donor variability is notable in UCB NK cells for all measured parameters

#### Cell expansion

The variability in fold expansion between donors was observable at each of the timepoints measured, and increased over time; days 7 (3.3-fold ±2.4), 14 (20.5-fold ±11.0) and 21 (62.2-fold ±35.4) of culture, despite each CBU undergoing the same process of isolation and culture (day-21 range from 13 – 144-fold; **Figure 2B**). Measurement of the total NK fold expansion achieved by UCB and APB cells after 21 days, indicated that there was no significant difference in expansion between the two cell sources, however APB NK cells displayed lower inter-donor variability (82.6-fold ±16.9; day-21 range from 57 – 102-fold) compared to UCB NK cells (p>0.05; **Figure 2C**), albeit the number of samples used in each group are different, which could influence the results. We noted that there was a positive correlation between day 7 and day 21 NK fold expansion (p<0.001; r=0.5896). Subsequent regression analysis supported a causative relationship between day 7 and day 21 NK fold expansion, thus highlighting the importance of the first 7 days of NK cell expansion in determining long term performance (p<0.01; R^2^ = 0.6504; **Figure 2D**). Furthermore, these results suggest that measurements of fold-expansion at early time points may be predictive of this measure at later time points (e.g. day-21).

#### Cytotoxic potential

Cytotoxic potential of the expanded UCB NK cells was quantified by measuring K562 target specific lysis at a range of effector to target cell ratios. We identified a high degree of inter-donor variability at each timepoint; days 7 (48.8% ± 15.4), 14 (42.2% ± 20.7), and 21 (44.1% ± 10.5; **Figure 2E**). Comparison of the profile of cytotoxicity between expanded APB and UCB NK cells in co-culture with the K562 cell line indicated that APB NK cells are significantly more cytotoxic than UCB NK cells at 2.5:1, 5:1 and 10:1 effector to target cell ratios under these expansion conditions, and have a lower standard deviation (10:1 K562 lysis was 68.6% ± 4.7; p<0.05; **Figure 2F**). Observations indicated that the differences between the two source materials increased as the relative proportion of effector cells increased in the co-culture. But this could be due to differences in the size of the cohort, or the slight differences in culture conditions.

Moreover, results showed that, as with NK fold expansion, there was a positive correlation between day 7 and day 21 K562 specific lysis (p<0.001; r=0.5936), and according to results from linear regression, day 7 K562 specific lysis could be a useful predictor of day 21 performance (p<0.01; R^2^ = 0.3524; **Figure 2G**). Thus, poor cytotoxic potential of UCB NK cells may be predictable early in our expansion system.

#### Phenotypic characterization

**Figure 3:**
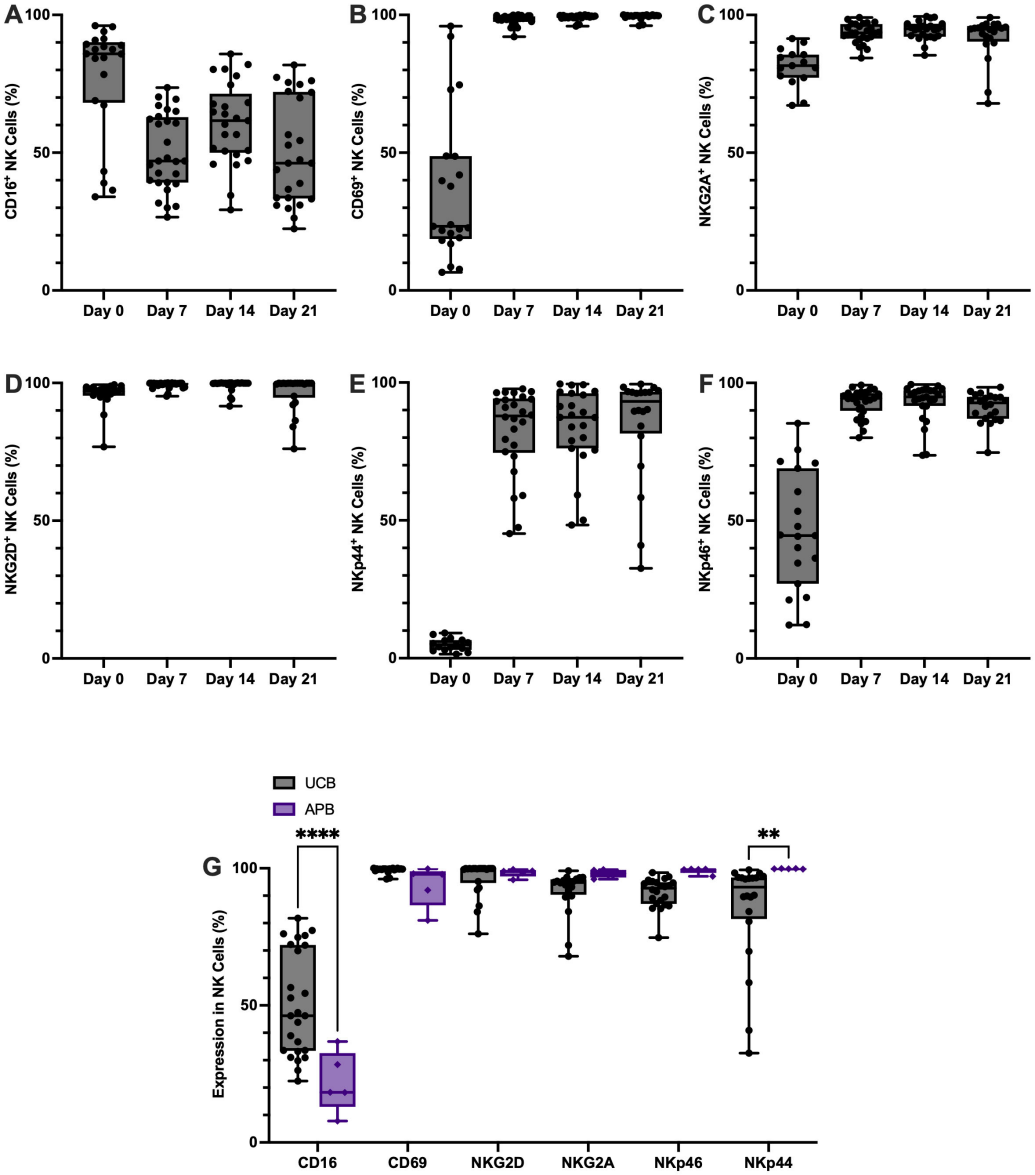
High proportions of NK cells expanded from CD3- CBMCs in a feeder free-expansion system express markers of NK cell activation and are phenotypically distinct from APB NK cells. NK cells (defined as CD3-CD56+) that had been expanded from CD3- CBMCs were flow cytometrically phenotyped regularly throughout expansion to measure the proportion that were positive for the following markers: **(A)** CD16, **(B)** CD69, **(C)** NKG2A, **(D)** NKG2D, **(E)** NKp44 and **(F)** NKp46 (n=25). **(G)** NK cell phenotype after 21 days of ex vivo expansion in a feeder free expansion system was compared between UCB (n=25) and APB (n=5) NK cells, results showed that there were significant differences between CD16 and NKp44 expression in cells isolated from the different starting materials. **p ≤ 0.01 and ****p ≤ 0.0001.

Phenotypic analysis of key NK cell markers at days 0, 7, 14, and 21 of expansion effectively tracked the activation of UCB NK cells during feeder free expansion (**Figures 3A–F**). NK cell activation markers such as, CD69, NKG2D, NKp44 and NKp46, increased over the course of expansion (**Figures 3B, D–F**, respectively). This was similar for the inhibitory receptor NKG2A, which is known to be highly expressed by UCB NK cells (17) (**Figure 3C**). The expression of activating receptor NKp44 also increased throughout expansion, however, the variability was higher between donors (84.9% ± 19.5 at day-21; **Figure 3E**). By contrast, CD16 expression in UCB NK cells decreased between isolation at day-0 and expansion at day-7 with some recovery by day-14 but with noticeable inter-donor variability at day-21 of expansion (50.5% ± 19.1; **Figure 3A**).

Results comparing the phenotype of expanded APB with UCB NK cells showed that CD69, NKG2D, NKG2A, and NKp46 expression was comparable in both groups (p>0.05; **Figure 3G**). The two NK cell markers which showed most inter-donor variability in UCB NK cells, CD16 and NKp44, were differentially expressed between APB and UCB NK cells (p<0.01; **Figure 3G**). CD16 expression was higher in UCB versus APB NK cells, and NKp44 expression was lower in UCB compared to APB NK cells, supporting existing observations that there are clear phenotypic differences in NK cell subtypes expanded from the different cell sources (17, 18).

### UCB donor and unit characteristics influence NK cell performance in *ex vivo* culture

**Figure 4:**
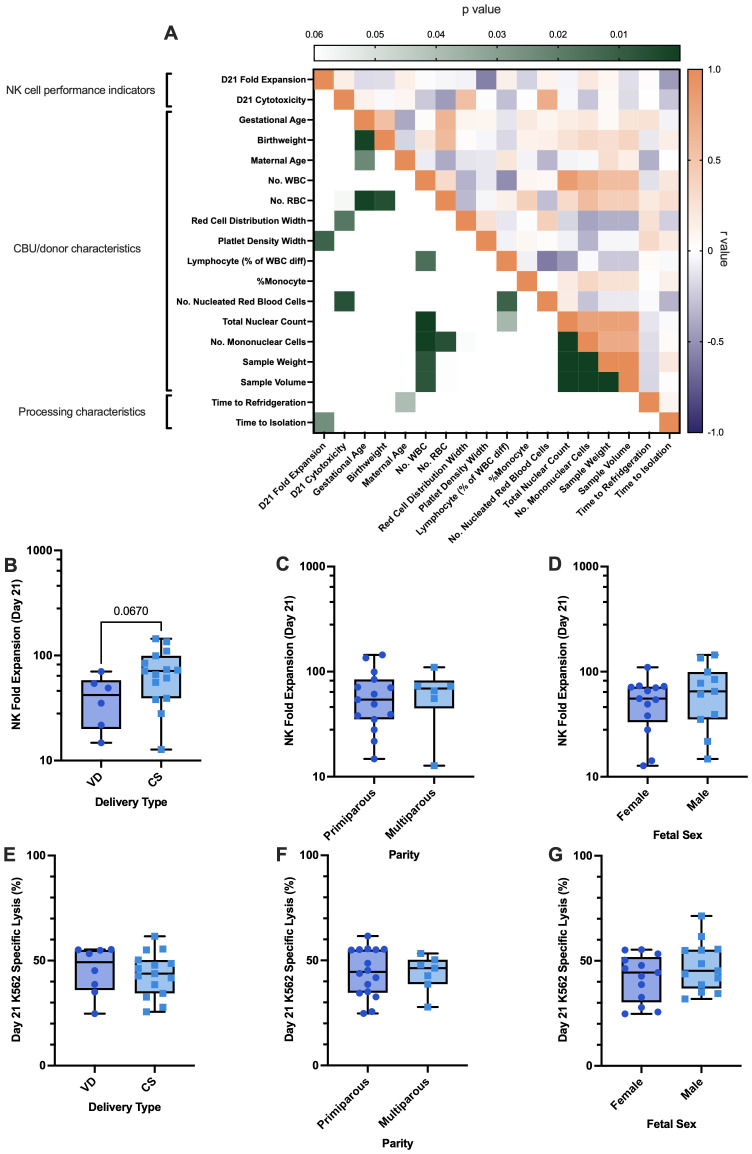
CBU/donor characteristics and variables in sample processing are associated with the ex vivo performance of UCB NK cells. **(A)** A correlational analysis matrix of the NK cell performance indicators for each sample (day-21 NK cell fold expansion and day-21 K562 specific lysis; n=25) and their CBU/donor characteristics or processing characteristics. Intensity of green for each comparison corresponds to degree of significance on the left-hand side of the heatmap. Whilst on the right-hand side, comparisons highlighted in orange correspond to positive relationships (r value), blue correspond to negative relationships, and shade intensity shows the degree of the relationship (all numerical p and r values in **Supplementary Figure 3**). **(B–G)** The impact of categorical CBU/donor characteristics on NK cell performance indicators (n=25). **(B)** Comparison of NK cell fold expansion between VD and CS deliveries indicated a trend for CS UCB NK cells to achieve a higher fold expansion ex vivo, although this did not reach statistical significance. **(C–G)** Delivery type had no impact on NK cell cytotoxicity, nor did parity or fetal sex have impacts on NK cell performance indicators.

Through our characterization of UCB-derived NK cells we observed substantial variability between the *ex vivo* performance of CD3^-^ CBMCs from different CBUs. Comparison with equivalent cells expanded from APB (albeit with a smaller cohort) also suggested that this effect may be more prevalent in UCB. To identify possible factors that may be related to this variance, correlational analysis was used to assess the relationships between *ex vivo* NK cell performance indicators and CB unit/donor characteristics (n=25; **Figure 4A**; **Supplementary Figure 4A**).

Results from this analysis identified a significant relationship between decreased platelet density width (PDW) and increased day-21 NK fold expansion (p<0.05; r=-0.582; **Figure 4A**), whilst increased red cell distribution width (RDW) and the number of nucleated red blood cells (NRBCs) correlated with day-21 target specific lysis against K562 (cytotoxicity; p<0.05; r=0.551; **Figure 4A**, p<0.01; R = 0.559; **Figure 4A** respectively).

UCB NK cell performance indicators were also compared between categorical factors relating to the UCB donor/unit, (n=25; **Figures 4B–G**). Whilst maternal parity and fetal sex were not associated with any effects on either NK cell fold expansion or cytotoxicity in our study (p>0.05), there was a trend for NK cells derived from CBU collected from caesarean sections (CS-both elective and emergency, see **Supplementary Figure 5** for breakdown) to achieve a higher fold expansion by day 21 of culture (72.7-fold ±37.5), compared to those isolated from CBU collected from vaginal deliveries (VD; 40.9-fold ±21.0), however this did not reach statistical significance in this cohort (p<0.07; **Figure 4B**; subtypes of delivery plotted in **Supplementary Figure 5A**).

### NK cell fold expansion can be enhanced by minimizing the time to isolation and adding a cryopreservation step

**Figure 5:**
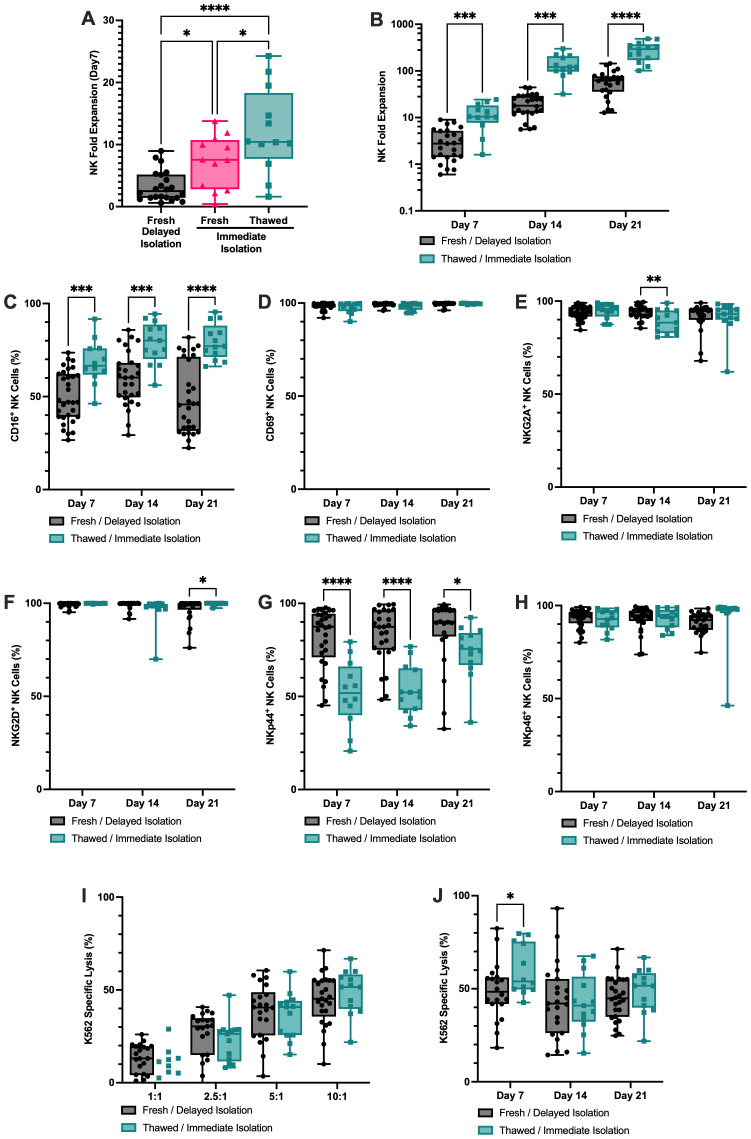
Cryopreserved CD3- CBMCs isolated <24h after collection provided the optimal starting material for enhanced NK cell fold expansion. **(A)** Cryopreserved CD3-CBMCs isolated immediately (<24h after CBU collection; n=13 green bars on all plots) achieved significantly higher NK fold expansion in 7-day ex vivo feeder free system compared to matched non-cryopreserved starting material (n=13)(pink bar) and unmatched CD3^-^ CBMCs where isolation was delayed (44h ±7.7 post collection; n=25 grey bars on all plots). **(B)** NK cell fold expansion continued to be higher from the optimal starting material (cryopreserved, immediately isolated CD3^-^ CBMCs) at days 14 and 21 of expansion, compared to non-optimal starting material (non-cryopreserved CD3^-^ CBMCs where isolation was delayed (unmatched CBUs). Flow cytometric characterization of NK cells throughout expansion showed that cells expanded from the different starting materials were phenotypically distinct **(C–H)**. Namely, CD16 was increased and NKp44 was decreased in NK cells expanded from the optimal starting material. **(I)** Measurement of day-21 K562 specific lysis is comparable between NK cells from the different starting materials at each E:T. **(J)** Over the course of expansion, NK cells expanded from the optimal starting material show increased K562 specific lysis at day 7 at 10:1 E:T. *p<0.05, ***p ≤ 0.001 and ****p ≤ 0.0001.

In addition to CBU/donor characteristics our results highlighted a significant relationship between time of CBU collection and CD3^-^ CBMC isolation, and subsequent NK fold expansion (p<0.05; r=-0.457; **Figure 4A**). To investigate this, we compared expansion characteristics between CD3^-^ CBMCs that were isolated <24h post UCB collection (19h ±3.3; termed immediate isolation; n=13) to cells isolated 44h ±7.7 post collection (termed delayed isolation; n=25). Following 7 days of expansion, immediately isolated, UCB NK cells demonstrated increased fold expansion (7.2-fold ±4.3) compared to NK cells from unmatched delayed isolation CD3^-^ CBMCs (3.3-fold ±2.5; p<0.05; **Figure 5A**).

Since banking cryopreserved isolated cells for future use would be highly desirable from a manufacturing point of view, we also tested NK cell expansion from immediately isolated CD3^-^ CBMCs that had been cryopreserved. Our results showed that CD3^-^ CBMCs that were immediately isolated and cryopreserved had significantly higher fold expansion (12.2-fold ±6.9) compared to unmatched delayed and, interestingly, also matched immediately isolated CD3^-^ CBMCs that were expanded without a cryopreservation step, using the same protocol within the first 7 days (3.3-fold ±2.5; p<0.05; **Figure 5A**).

When UCB NK cells from the “optimal starting material” (cryopreserved CD3^-^ CBMCs isolated immediately) were expanded in culture for 21-days, the fold expansion was significantly higher compared to cells expanded in the same system from non-optimal starting material (non-cryopreserved matched CD3^-^ CBMCs; delayed isolation-unmatched units) at each of the timepoints tested, day-7 (12.2-fold ±6.9; p<0.001), 14 (142.8-fold ±74.24; p<0.001) and 21 (283.8-fold ±126.5; range from 101 – 487-fold; p<0.0001; **Figure 5B**). Phenotypically these two groups had similar expression of CD69 and NKp46 (**Figures 5D–H**). However, expanded NK cells from the optimal starting material had higher expression of CD16 (**Figure 5C**), and NGK2D (**Figure 5F**), but lower expression of NKG2A (**Figure 5E**) and NKp44 (**Figure 5G**), with the latter statistically significant at all time points.

Despite the differences in expansion and phenotype between the two processing modalities, the cytotoxicity demonstrated by NK cells against the K562 cell line were comparable at each E:T ratio at day-21 (p>0.05; **Figure 5I**). Although, NK cells expanded from the optimal starting material showed higher K562 specific lysis at day 7 (p<0.05), by days 14 and 21 it was comparable between the two groups (at 10:1 effector-to-target ratio; **Figure 5J**).

### Once corrected for time to cell isolation, the relative contribution of other parameters changes

**Figure 6:**
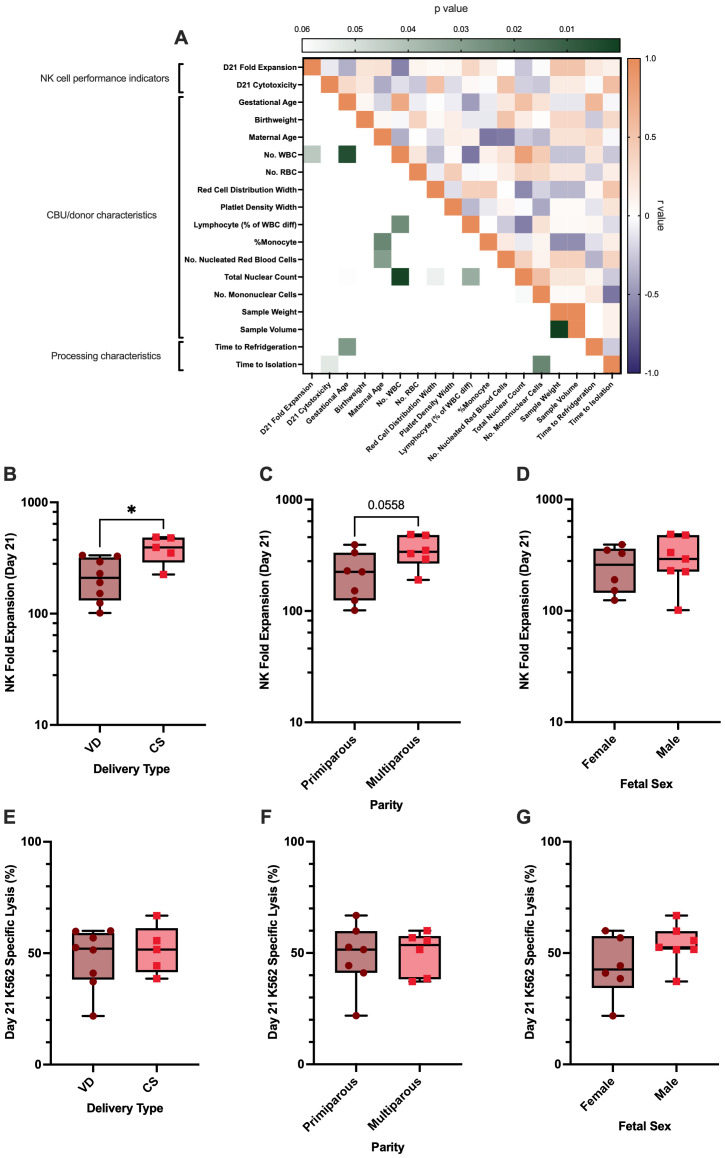
Cryopreserved CD3- CBMCs isolated in <24h from CS deliveries demonstrated increased NK cell fold expansion in feeder free culture compared to VDs, other CBU characteristics are not related to NK cells performance. A correlational analysis matrix of the NK cell performance indicators for each sample (day-21 NK cell fold expansion and day-21 k562 specific lysis; n=13) and their CBU/donor characteristics or processing characteristics. Intensity of green for each comparison corresponds to degree of significance on the left-hand side of the heatmap. Whilst on the right-hand side, comparisons highlighted in orange correspond to positive relationships (r value), blue correspond to negative relationships, and shade intensity shows the degree of the relationship (all numerical p and r values in supplementary 2). The influence of categorical CBU/donor characteristics on NK cell performance indicators such as NK cell fold expansion and K562 specific lysis, when using the optimal starting material (cryopreserved CD3^-^ CBMCs isolated in <24h; n=13). **(B)** NK cell fold expansion was higher in cells isolated from CBUs collected from CS compared to VD. **(C)** There was a trend for increased NK cell fold expansion in cells from multiparous compared to primiparous pregnancies, however this did not reach statistical significance. **(D–G)** Delivery type and parity did not affect NK cell cytotoxicity, nor did fetal sex affect either of the NK cell performance indicators.

A repeat of the correlational analysis between *ex vivo* NK cell performance indicators and CBU/donor characteristics was conducted, this time on units isolated immediately (<24h) and cryopreserved before expansion. Results show that despite PDW, RDW and NRBCs showing similar variability in this cohort (**Supplementary Figure 4B**), these characteristics no longer significantly affect parameters of NK cell performance in this group (p>0.05; **Figure 6A**). This finding suggests that prolonged extracorporeal exposure to red blood cells and platelets, particularly when activated, may impair NK cell performance, and that early isolation can mitigate this effect.

Additionally, once we corrected for the influence that time to isolation had on cell quality, we found that CBUs obtained from CS, expanded significantly better (387.2 ± 107.5 fold) than those from VD (219.1 ± 91.66 fold; p=0.0117; **Figures 6A, B**). The specific K562 lysis capacity (cytotoxicity), however, was equivalent (**Figure 6E**). A trend for CBUs from multiparous births to expand better was also observed but did not reach statistical significance (**Figure 6C**). Fetal sex had no influence on either expansion or specific lysis, regardless of time to isolation (**Figures 6D, G**).

### Performance capability of UCB NK cells appears to be a cell intrinsic property and is independent of the isolation method or expansion system used

**Figure 7:**
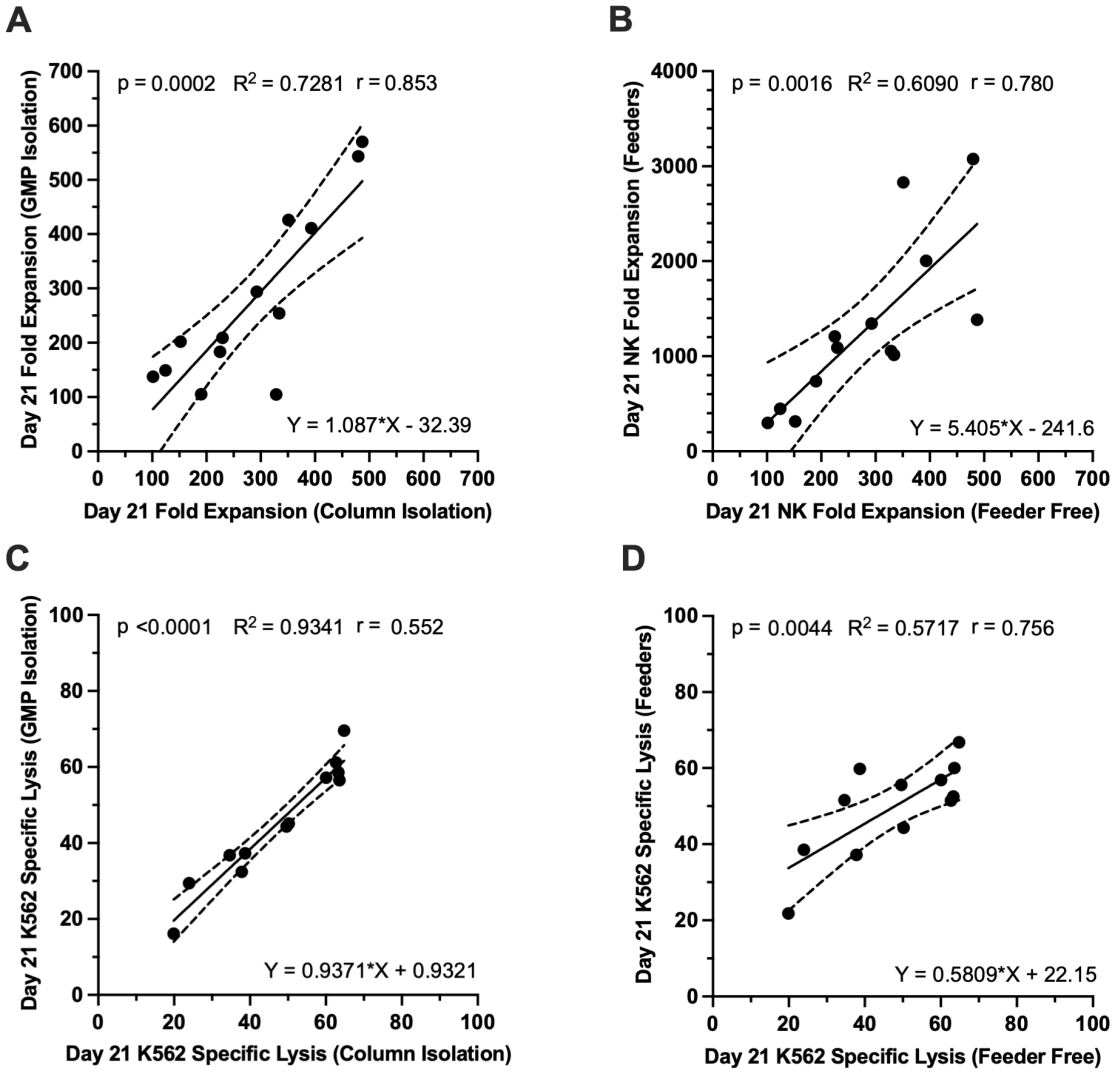
Performance capacity for CD3-CBMC-derived NK cells is consistent between different isolation strategies and different expansion protocols, suggesting that these properties are largely intrinsic to the starting cells and could be identified through small-scale batch testing of cells. (**A, B**) Day 21 NK cell fold expansion and K562 specific lysis from CD3^-^CBMCs starting material isolated using column-based research grade isolation was predictive of performance of matched CD3^-^CBMCs isolated by GMP compliant FACS. **(C, D)** Day 21 NK cell fold expansion and K562 specific lysis from CD3^-^CBMCs starting material grown in the basic feeder free culture system was also predictive of the performance of matched cells that were expanded ex vivo in a feeder cell-based culture system. **(A–D)** Measured via correlation and linear regression analysis (solid line; dashed lines show 95% confidence intervals). *p<0.05, **p ≤ 0.01, ***p ≤ 0.001.

Although the data presented thus far was collected from experiments where CD3^-^ CBMCs were isolated using a manual magnetic bead/column system (QuadroMACS separator, Miltenyi Bio), results from NK cell expansions conducted in parallel using a GMP compliant closed isolation process based on fluorescence activated cell sorting (Tyto, Miltenyi Bio) found that CD3^-^ CBMC starting material from matched UCB units, behaved similarly (p<0.001; R^2^ = 0.7281; **Figure 7A**). Furthermore, NK cell fold expansion from CD3^-^ CBMC starting material that was grown in basic feeder free culture medium was predictive of NK cell fold expansion performance in matched CBUs expanded using a feeder cell expansion system, even if the absolute numbers were different (P<0.01; R^2^ = 0.6090; **Figure 7B**). This was also true for day 21 specific lysis of K562 cells by NK cells expanded in either system (**Figures 7C, D**).

### Post-expansion cryopreservation does not affect UCB NK cell characteristics

**Figure 8:**
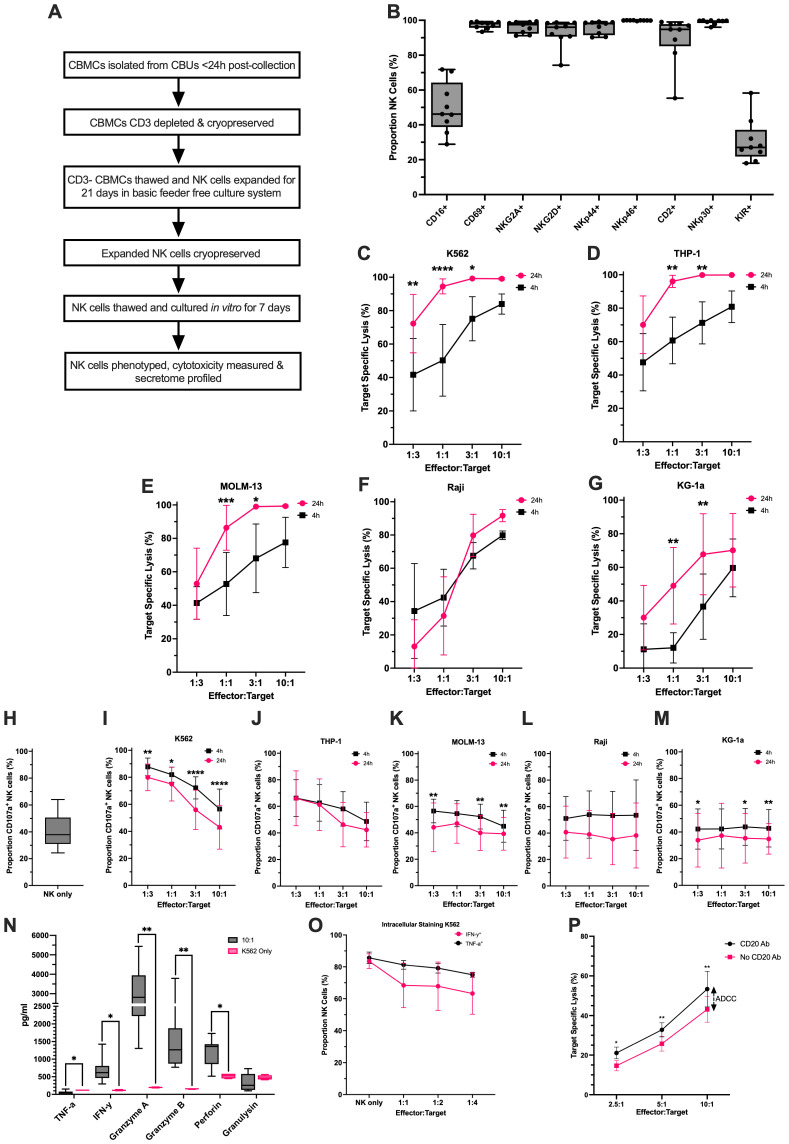
Ex vivo expanded UCB NK cells survive cryopreservation and remain functionally active in in vitro cytotoxicity assays. **(A)** UCB NK cells were expanded from cryopreserved CD3^-^ CBMCs, isolated <24h after collection. Cells were expanded ex vivo in the feeder free culture system and at day-21 they were cryopreserved. **(B)** Thawed NK cells demonstrated an activated mature NK cell phenotype (n = 8). **(C – G)** The cells were functionally active and specifically lysed five different cancer cell target lines (K562, THP-1, MOLM-13, Raji and KG-1a) in 4h and 24h co-cultures, however, the percentage of specific lysis was variable especially at lower E:T ratios (n = 7). **(H–M)** CD107a expression by NK cells was also measured as a marker for degranulation in NK only cultures and in the co-culture assays with each cancer cell line (n=7). **(N)** Secretome analysis of the 10:1 NK cell-to-K562 co-cultures indicated that when stimulated the NK cells secrete increased levels of Granzyme A, Granzyme B, Perforin and IFN-y compared to the K562 only control, however the TNF-a secretion is higher in the K562 only control (n=7; p<0.05). **(O)** Intracellular staining of IFN-y and TNF-a confirmed their production by NK cells when cultured with and without K562 (n=3). **(P)** Increased lysis of Raji cells by NK cells in the presence of a CD20 antibody compared to the no CD20 antibody control shows that the ex vivo expanded UCB NK cells are also capable of ADCC (n=3; p<0.05). Created in BioRender. https://BioRender.com/0sr99is. *p<0.05, **p ≤ 0.01, ***p ≤ 0.001 and ****p ≤ 0.0001.

Following th*e ex vivo* expansion described above, cells from 8 of the 13 CBUs used above and not used for analysis were cryopreserved and maintained in liquid nitrogen for approximately 2 years. These cells were then thawed and cultured for 7 days in our basic culture medium, before being re-analyzed for phenotypic and functional characteristics. **Figure 8** depicts the process followed (a) and the cell phenotype (b), which is comparable to the cells prior to cryopreservation, with high expression of an extended panel of mature activating receptors. Crucially the NK cells retained their capacity to lyse not only K562 cells (**Figure 8C**), but also a further four leukemic cell lines of different origins (THP-1 (7D) MOLM13 (7E), RAJI (7F) and KG1a (7G)) at varying effector to target ratios over short (4h) and longer (24h) co-culture times. The killing capacity was accompanied by increased expression of CD107a from a mean of 40.4 ± 1 3% in NK cells prior to co-culture to a high of 74.58 ± 13.6 in co-culture with K562 at low E:T ratios (**Figures 8H–M**) and secretion of Granzyme A and B, Granulysin and INFγ (**Figure 8N**). Intracellular staining confirmed increased INFγ and TNFα content in NK cells (**Figure 8O**). We also showed that these NK cells are capable of antibody dependent cell mediated cytotoxicity (ADCC) with significantly enhanced killing of Raji lymphoma cells in the presence of the anti-CD20 antibody Obinutuzumab (**Figure 8P**).

### Proposed strategy for UCB NK cell production to maximize potential and reduce cost of goods

Based on the findings of our study, we propose a workflow for the production of NK cell therapeutic products which enables preselection of the highest quality CBUs with early batch testing, thus minimizing wastage and cost of production (**Figure 1**). This workflow relies on close liaison with the UCB bank or UCB supplier but eliminates the variability and time sensitivity associated with the processing of fresh units as well as the challenges associated with depleting T cells from frozen units. Utilizing the selection criteria which we identified, UCB units can be immediately diverted to a processing lab, where CBMCs can be isolated, CD3 depleted and cryopreserved at different volumes (cell numbers). Small aliquots from each batch can then be used for miniaturized culture and assessment of expansion potential and cytotoxicity within as little as 5–7 days. Thus, starting material qualification can be achieved in small volumes at lower cost before investing in the expansion of a whole unit. Following this derisking, the most highly proliferative, and cytotoxic units can then be chosen for large scale expansion and clinical manufacturing. This constitutes a novel approach to the processing of starting material and allows a qualification step, prior to investing in expansion of a whole UCB unit.

## Discussion

Adoptive cell immunotherapies represent a paradigm shift in the treatment of cancer. Harnessing the power of the immune system has revolutionized the treatment of malignancies. However, despite the clinical success of CAR-T therapies, this is largely restricted to B cell malignancies and patient access is still limited. The autologous nature of the treatment along with high costs of production and tight manufacturing timelines, as well as the requirement for delivery in specialized treatment centers, are all major factors impacting access. Therefore, the use of allogeneic cell sources could alleviate some of these hurdles. NK cell therapies have emerged as an attractive alternative to T-cell immunotherapy because they have several advantages both in terms of procurement and manufacture, as well as their innate killing capacity. Importantly, NK cells can be used from allogeneic healthy individuals without the risk of immune rejection or GvHD. This is especially true of UCB derived NK cells (19) with the added bonuses that they are also less likely to carry infections and are readily available with no donor attrition or added donor burden.

However, to be clinically useful UCB derived NK cells must undergo a manufacturing process involving extensive expansion and activation, the first step of which is the selection and isolation of the starting material (the cells). Given the challenge of inherent inter-donor variability, this first step in the selection and qualification of starting material is crucial. In this study we assessed the expansion and cytotoxicity potential of NK cells derived from fresh UCB and retrospectively correlated these results with both donor and processing characteristics, thereby establishing criteria enabling the selection of optimal units and adopting processing parameters most likely to produce a clinically effective product. Furthermore, we have proven that both starting material and end product can be successfully cryopreserved without loss of functional activity (*in vitro* cytotoxicity), thus facilitating the logistics of manufacture and delivery at a clinical scale. Utilizing fresh UCB units and introducing a cryopreservation step after cell selection but before expansion is, as far as we know, novel, and it provides a unique solution to the complex logistics of production of UCB-derived NK cells for therapy. Further, we demonstrate that these cells have higher expansion potential than cells from matched units which were not cryopreserved following isolation, at least in the first 7 days of culture.

By systematically isolating NK cells from over 30 individual fresh CBUs, we established that the parameter with the greatest influence in NK cell performance was time to isolation (r=-4.57, p<0.05). Cells isolated from fresh cords within 24 hours of collection far outperformed those with delayed isolation (~44h) in fold expansion without compromising cytotoxicity. This supports observations by Marin et al. (2024), where <24h CBU processing time was a significant predictor of superior outcome from CAR-NK cell therapy recipients (7), though their study used cryopreserved CBUs as a starting material, while our study uses fresh whole blood for initial cell isolation followed by a cryopreservation step of CD3 depleted CBMCs, avoiding isolating cells from cryopreserved whole blood.

The enhanced fold expansion of cells from fresher CBUs is likely linked to the decreased exposure of NK cells to whole blood storage lesions, for example the elevated levels of activated platelets that accumulate at standard cold holding temperatures (20). Activated platelets have a higher PDW and are known suppressors of NK cell activation and function through several pathways including secretion of the immunosuppressive cytokine TGF-β (21, 22). This could explain why initially CBU PDW was negatively correlated with expansion in delayed isolated cells but became less significant once time to isolation was decreased.

Similar relationships were observed between erythrocyte characteristics in pre-processed CBUs and NK cell performance for CBUs where isolation was delayed, but which became insignificant when time to isolation was <24 hours. We found that NK cell cytotoxicity was positively correlated with numbers of NRBCs and RDW. Such measurements are usually indicative of adverse intrauterine environment such as hypoxia, due to birth complications, and may be linked with associated inflammation in UCB (23). Elevated NRBCs have recently been proposed as a quality parameter for CBU selection for HCT due to their association with greater engraftment capacity (24). However, in the recent CD19 CAR-NK clinical trial by Marin et al. (2024) receiving CAR-NK cells from CBUs with ≤8 × 10^7^ NRBCs was found to be a predictor of better outcomes in patients with CD19^+^ B cell malignancies (7). Further *in vitro* studies found that CAR-NK cells from CBUs with NRBCs above the 8 × 10^7^ threshold demonstrated reduced cytolytic activity (7) this contrasts with the relationship identified here. However, it is important to note that the study above used cryopreserved whole cord blood as a source of NK cells. Our studies have all been carried out in fresh UCB units, which may have an impact on the importance of red blood cell parameters for eventual NK cell performance. Other factors which can also affect platelet and erythrocyte activation, function and viability include the UCB storage containers, anticoagulant and collection technique used (25). We chose to use fresh UCB rather than cryopreserved units for both logistical as well as processing issues, though we understand that this may not be the commonest source in other sites.

When we analyzed all cord blood units together, we saw a trend in the influence of delivery mode on NK cell expansion. However, when we controlled for processing time by analyzing only units processed within 24 hours, cells isolated from CBUs from CS deliveries achieved superior NK cell fold expansion compared to those from VDs, without compromising on cytotoxic potential *in vitro*, with the effect becoming statistically significant (P<0.05). This indicates that the powerful negative effect of delayed isolation had previously masked the significant impact of delivery mode on NK cell growth potential.

It is unclear why NK cells from CS CBUs are more proliferative *ex vivo*, however, the absence of the transmission of microbes from the maternal birth canal to the neonate in CS deliveries could potentially alter CBU cellular profiles and NK cell activation. Since, leukocyte count, profiles and cytokine secretion are known to be altered in UCB from CS versus those from VDs (26–28).

Importantly, our results also show that despite the widely reported loss of cytotoxicity in freeze/thawed NK cells (29), the functional phenotype of NK cells expanded *ex vivo* from cryopreserved CD3^-^ CBMCs in our hands is comparable to cells that were expanded from matched fresh (non-cryopreserved) starting material. In fact, cryopreserved pre-expanded NK cells do not lose their cytotoxicity post-thaw even against difficult to kill cell lines such as KG-1a. Above all, we show that cryopreserved CD3^-^ CBMCs provide an optimal starting material for obtaining high yields of relatively pure NK cells following *ex vivo* expansion. This enhanced proliferation may be due to changes in the heterogenous population of cells that make up CD3^-^ CBMCs post-thaw; for example, the preferential loss of granulocytes during thawing, which can suppress the activation of NK cells (30–32). While we did not specifically stain for granulocyte populations in the fresh cells prior to cryopreservation, flow cytometry analysis (FSC/SSC) of the CD3 depleted cells before and after cryopreservation revealed a shift in the leukocyte composition. This included a distinct reduction in the population typically corresponding to monocytes in the post thaw samples, which is even more pronounced after 7 days of culture (**Supplementary Figure 7**).

In addition to the performance benefits of this cryopreserved starting material, it also presents numerous logistical benefits that would streamline the manufacturing of an allogeneic NK cell therapy. The ability to transport and store multiple batches of cryopreserved ready to expand staring material is particularly advantageous in scenarios like ours, where the manufacturing facilities for large scale-expansion are not close to the UCB collection/processing center. Considering the time sensitivity of starting material isolation (<24h), this also reduces the risk of shipping delays having a detrimental effect on product quality. Use of cryopreserved cells also permits more flexibility in the scheduling of NK cell manufacturing in clean room facilities, an advantage to which the benefits have already been recognized in the utilization of cryopreserved leukapheresis material in autologous T cell therapy (33). Though this scenario requires the banking facility to have GMP compliant cell isolation facilities, this is not impossible to achieve. We utilized manual MACS isolation, but this is comparable to the Prodigy (Miltenyi Biotech) which utilizes identical technology in a closed system which is used routinely in clinical settings.

Additionally, cryopreservation of isolated cells enables the incorporation of a batch testing step within the manufacturing pipeline; a small-scale evaluation of NK cell performance indicators in basic feeder free expansion media could then be used to select the best units to bring forward to large-scale expansion. The batch testing of cells from each CBU and selection of highest performing units for downstream manufacturing would enhance standardization of the efficacy of the cellular therapy in line with GMP guidelines. Forward planning and synchronization of numerous expansions enabled through cryopreservation of the starting material would also reduce the costs of GMP-compliant cleanroom facilities usage by reducing the number of days required for manufacturing. For example, recent transcriptomic analysis of UCB hematopoietic stem cell-derived NK cells by van Vliet et al. (2024) developed a predictive tool that uses multi-factorial gene signatures (such as upregulated Granzyme B and Perforin) that could serve as criteria for selection for units with enhanced cytotoxicity (34). This type of batch testing is not feasible when cells are cultured immediately from fresh UCB or when cryopreserved CBUs are used as once the unit is thawed, it must be processed and all of the isolated cells expanded immediately (as in Marin et al., 2024 (7)). Selection of the best performing CBUs could effectively address the difficulties that the observed inter-donor variability causes, as well as provide an early and precise indication of overall yield per unit, to minimize the wastage of resources on underperforming units. Moreover, this workflow allows the utilization of CBUs which may not reach banking thresholds for transplant purposes, but would still yield sufficient starting cells for NK cell manufacture.

In conclusion, the findings from this investigation suggest that cryopreserved CD3^-^ CBMCs isolated within 24h of UCB collection from CS deliveries are an optimal starting material for the development of UCB-derived NK cell immunotherapies. Using a simple feeder free culture system, with these optimal cords as starting material we generated on an average 1.14 x 10^9^ NK cells post expansion per 1x10^8^ CBMCs (**Supplementary Figure 6**). Using an optimized, feeder cell-based expansion procedure much greater expansion is likely. Likewise, cytotoxicity assays demonstrated potent cytotoxicity of expanded NK cells, nonetheless, strategies such as gene editing could be utilized to further enhance their cytotoxicity. Taken together, our proposed strategy for CBU selection, starting material cryopreservation and small-scale batch testing could substantially reduce wastage of resources on low performing units, and maximize the number of doses and product potency when combined with an optimized expansion system, thus ensuring the selection of the best possible starting material for large scale manufacturing.

## Data Availability

The original contributions presented in the study are included in the article/**Supplementary Material**. Further inquiries can be directed to the corresponding author.

## References

[1] Lodoen MB Lanier LL . Natural killer cells as an initial defense against pathogens. Curr Opin Immunol. (2006) 18:391–8. doi: 10.1016/j.coi.2006.05.002, PMID: 16765573 PMC7127478

[2] Liu S Galat V Galat Y Lee YKA Wainwright D Wu J . NK cell-based cancer immunotherapy: from basic biology to clinical development. J Hematol Oncol. (2021) 14:7. doi: 10.1186/s13045-020-01014-w, PMID: 33407739 PMC7788999

[3] Smyth MJ Cretney E Kelly JM Westwood JA Street SEA Yagita H . Activation of NK cell cytotoxicity. Mol Immunol. (2005) 42:501–10. doi: 10.1016/j.molimm.2004.07.034, PMID: 15607806

[4] Fang F Xie S Chen M Li Y Yue J Ma J . Advances in NK cell production. Cell Mol Immunol. (2022) 19:460–81. doi: 10.1038/s41423-021-00808-3, PMID: 34983953 PMC8975878

[5] Shanley M Daher M Dou J Li S Basar R Rafei H . Interleukin-21 engineering enhances NK cell activity against glioblastoma via CEBPD. Cancer Cell. (2024) 42:1450–1466.e11. doi: 10.1016/j.ccell.2024.07.007, PMID: 39137729 PMC11370652

[6] Hibler W Merlino G Yu Y . CAR NK cell therapy for the treatment of metastatic melanoma: potential & Prospects. Cells. (2023) 12:2750. doi: 10.3390/cells12232750, PMID: 38067178 PMC10706172

[7] Marin D Li Y Basar R Rafei H Daher M Dou J . Safety, efficacy and determinants of response of allogeneic CD19-specific CAR-NK cells in CD19+ B cell tumors: a phase 1/2 trial. Nat Med. (2024) 30:772–84. doi: 10.1038/s41591-023-02785-8, PMID: 38238616 PMC10957466

[8] Huppert V . Umbilical cord blood NK cells offer multiple advantages for cancer immunotherapy: lessons learned from Glycostem’s orphan drug oNKord^®^. Cell Gene Ther Insights. 07:1795–805. (2021).

[9] Mehta RS Shpall EJ Rezvani K . Cord blood as a source of natural killer cells. Front Med (Lausanne). (2016) 2:93/abstract. doi: 10.3389/fmed.2015.00093/abstract, PMID: 26779484 PMC4700256

[10] Laskowski TJ Biederstädt A Rezvani K . Natural killer cells in antitumour adoptive cell immunotherapy. Nat Rev Cancer. (2022) 22:557–75. doi: 10.1038/s41568-022-00491-0, PMID: 35879429 PMC9309992

[11] Kundu S Gurney M O’Dwyer M . Generating natural killer cells for adoptive transfer: expanding horizons. Cytotherapy. (2021) 23:559–66. doi: 10.1016/j.jcyt.2020.12.002, PMID: 33431318

[12] Maia A Tarannum M Romee R . Genetic manipulation approaches to enhance the clinical application of NK cell-based immunotherapy. Stem Cells Trans Med. (2024) 13:230–42. doi: 10.1093/stcltm/szad087, PMID: 38142460 PMC10940834

[13] Gurney M Kundu S Pandey S O’Dwyer M . Feeder cells at the interface of natural killer cell activation, expansion and gene editing. Front Immunol. (2022) 13. doi: 10.3389/fimmu.2022.802906, PMID: 35222382 PMC8873083

[14] Alnabhan R Madrigal A Saudemont A . Differential activation of cord blood and peripheral blood natural killer cells by cytokines. Cytotherapy. (2015) 17:73–85. doi: 10.1016/j.jcyt.2014.08.003, PMID: 25248279

[15] Wu X Zhang Y Li Y Schmidt-Wolf IGH . Improvements in flow cytometry-based cytotoxicity assay. Cytometry Part A. (2021) 99:680–8. doi: 10.1002/cyto.a.24242, PMID: 33068327

[16] Hosseini E Ghasemzadeh M Kamalizad M Schwarer AP . Ex vivo expansion of CD3depleted cord blood-MNCs in the presence of bone marrow stromal cells; an appropriate strategy to provide functional NK cells applicable for cellular therapy. Stem Cell Res. (2017) 19:148–55. doi: 10.1016/j.scr.2017.01.010, PMID: 28171825

[17] Luevano M Daryouzeh M Alnabhan R Querol S Khakoo S Madrigal A . The unique profile of cord blood natural killer cells balances incomplete maturation and effective killing function upon activation. Am Soc Histocompatibility Immunogenetics. (2012) 73:248–57. doi: 10.1016/j.humimm.2011.12.015, PMID: 22234167

[18] Buckle I Johnson A Rojas IL Weinert V Sester DP Radford K . High dimensional analysis reveals distinct NK cell subsets but conserved response to stimulation in umbilical cord blood and adult peripheral blood. Eur J Immunol. (2023) 53:e2250118. doi: 10.1002/eji.202250118, PMID: 37025016

[19] Liu E Marin D Banerjee P Macapinlac HA Thompson P Basar R . Use of CAR-transduced natural killer cells in CD19-positive lymphoid tumors. New Engl J Med. (2020) 382:545–53. doi: 10.1056/NEJMoa1910607, PMID: 32023374 PMC7101242

[20] Ayukawa O Nakamura K Kariyazono H Ikeda R Arima J Shinkawa T . Enhanced platelet responsiveness due to chilling and its relation to D40 ligand level and platelet-leukocyte aggregate formation. Blood Coagulation Fibrinolysis. (2009) 20:176–84. doi: 10.1097/MBC.0b013e328322ffd5, PMID: 19300046

[21] Du Y Liu X Guo SW . Platelets impair natural killer cell reactivity and function in endometriosis through multiple mechanisms. Hum Reproduction. (2017) 32:794–810. doi: 10.1093/humrep/dex014, PMID: 28184445

[22] Viel S Marçais A Souza-Fonseca Guimaraes F Loftus R Rabilloud J Grau M . TGF-b inhibits the activation and functions of NK cells by repressing the mTOR pathway. Sci Signal. (2016) 9:ra19–9. doi: 10.1126/scisignal.aad1884, PMID: 26884601

[23] Hebbar S Misha M Rai L . Significance of maternal and cord blood nucleated red blood cell count in pregnancies complicated by preeclampsia. J Pregnancy. (2014) 2014:496416. doi: 10.1155/2014/496416, PMID: 24734183 PMC3964768

[24] Kondo AT Alvarez KCA Cipolletta ANF Sakashita AM Kutner JM . Nucleated red blood cell: a feasible quality parameter of cord blood units. Hematol Transfus Cell Ther. (2024) 46:221–7. doi: 10.1016/j.htct.2023.01.009, PMID: 36935342 PMC11221323

[25] Vani R Soumya R Manasa K Carl H . Storage lesions in blood components. Oxid Antioxid Med Sci. (2015) 4:125. doi: 10.5455/oams.130915.rv.019

[26] Werlang ICR Mueller NT Pizoni A Wisintainer H Matte U Martins Costa SH de A . Associations of birth mode with cord blood cytokines, white blood cells, and newborn intestinal bifidobacteria. PLoS One. (2018) 13:e0205962. doi: 10.1371/journal.pone.0205962, PMID: 30388115 PMC6214518

[27] Ly NP Ruiz-Pérez B Onderdonk AB Tzianabos AO Litonjua AA Liang C . Mode of delivery and cord blood cytokines: A birth cohort study. Clin Mol Allergy. (2006) 4:13. doi: 10.1186/1476-7961-4-13, PMID: 17002791 PMC1592116

[28] Thysen AH Larsen JM Rasmussen MA Stokholm J Bønnelykke K Brisgaard H . Prelabor cesarean section bypasses natural immune cell maturation. J Allergy Clin Immunol. (2015) 136:1120–1123.e4. doi: 10.1016/j.jaci.2015.04.044, PMID: 26094084

[29] Damodharan SN Walker KL Forsberg MH McDowell KA Bouchlaka MN Drier DA . Analysis of ex vivo expanded and activated clinical-grade human NK cells after cryopreservation. Cytotherapy. (2020) 22:450–7. doi: 10.1016/j.jcyt.2020.05.001, PMID: 32536506 PMC7387178

[30] Boonlayangoor P Telischi M Boonlayangoor S Sinclair TF Millhouse EW . Cryopreservation of human granulocytes: study of granulocyte function and ultrastructure. Blood. (1980) 56:237–45., PMID: 6249429

[31] Li P Lu M Shi J Hua L Gong Z Li Q . Dual roles of neutrophils in metastatic colonization are governed by the host NK cell status. Nat Commun. (2020) 11:4387. doi: 10.1038/s41467-020-18125-0, PMID: 32873795 PMC7463263

[32] Palano MT Gallazzi M Cucchiara M De Lerma Barbaro A Gallo D Bassani B . Neutrophil and natural killer cell interactions in cancers: Dangerous liaisons instructing immunosuppression and angiogenesis. Vaccines. (2021) 9:1488. doi: 10.3390/vaccines9121488, PMID: 34960234 PMC8709224

[33] Adriaansen J Stanton J Schaut W Bowden R . Compliance and cost control for cryopreservation of cellular starting materials: An industry perspective. Cytotherapy. (2022) 24:750–3. doi: 10.1016/j.jcyt.2022.02.004, PMID: 35304076

[34] van Vliet AA van den Hout MGCN Steenmans D Duru AD Georgoudaki AM de Gruijl TD . Bulk and single-cell transcriptomics identify gene signatures of stem cell-derived NK cell donors with superior cytolytic activity. Mol Therapy: Oncol. (2024) 32:200870. doi: 10.1016/j.omton.2024.200870, PMID: 39346765 PMC11426129

