# Supplementary material for: Umbilical cord blood natural killer cells for adoptive immunotherapy: identifying optimal starting material and processing parameters
Source: Front Immunol. 2026 Jan 13;16:1734453. doi: 10.3389/fimmu.2025.1734453 (PMC12835214; doi:10.3389/fimmu.2025.1734453)

Supplementary Figure 1. Flow cytometry gating strategy for flow cytometry-based cytotoxicity assay. The number of live target cells was determined by gating on the CTV+L/D ZombieNIR- population of cells. CD107a expression of NK cells was measured by gating on live, CTV-, CD3-CD56+ cells.

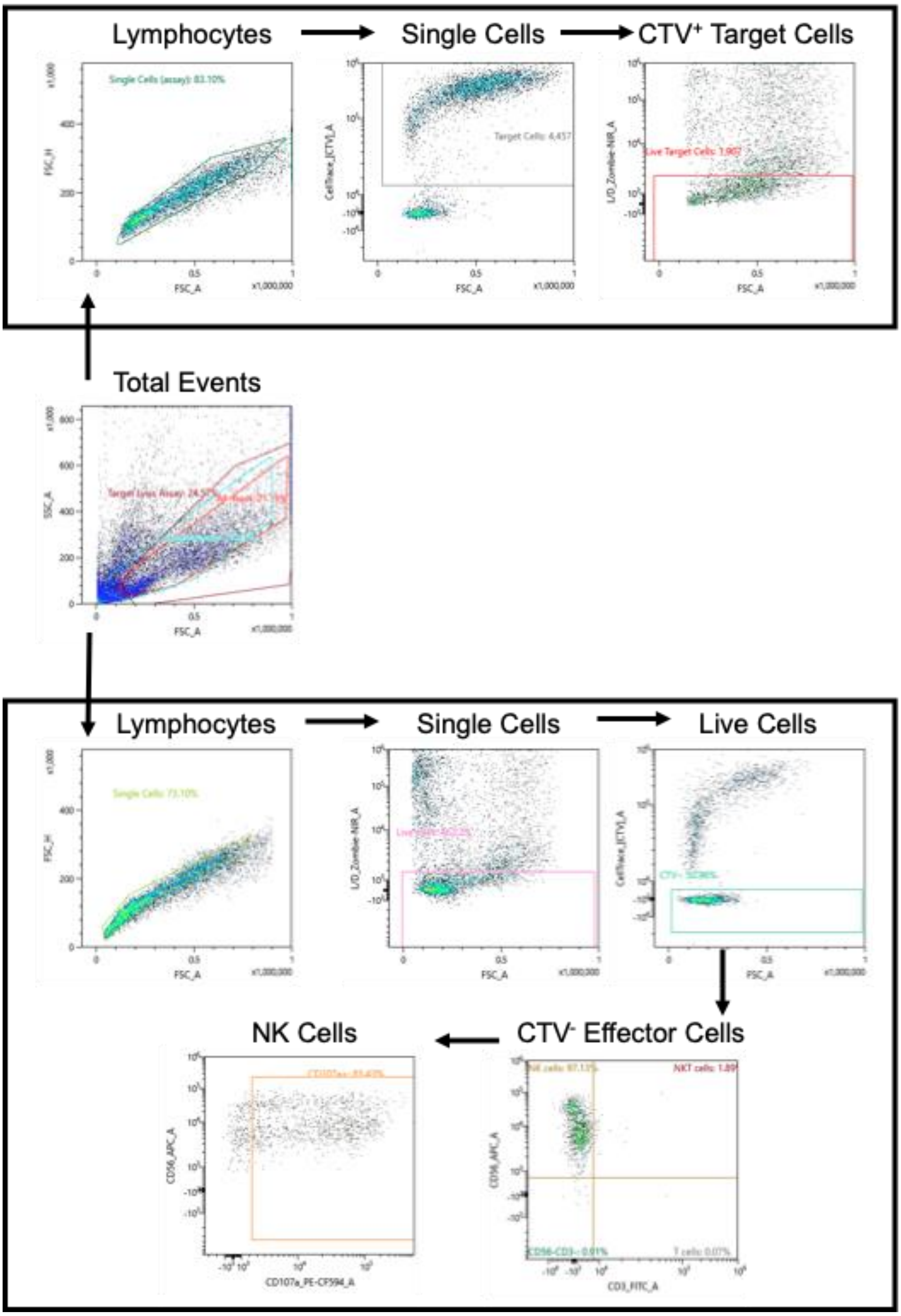

Supplementary Figure 2. Flow cytometry gating strategy for NK cell phenotyping panels. NK cells were gated on as the CD3-CD56+ cells from the live population of singlet lymphocytes, and expression of activating and inhibitory receptors was calculated as the percentage of CD3-CD56+ cells expressing a given marker.

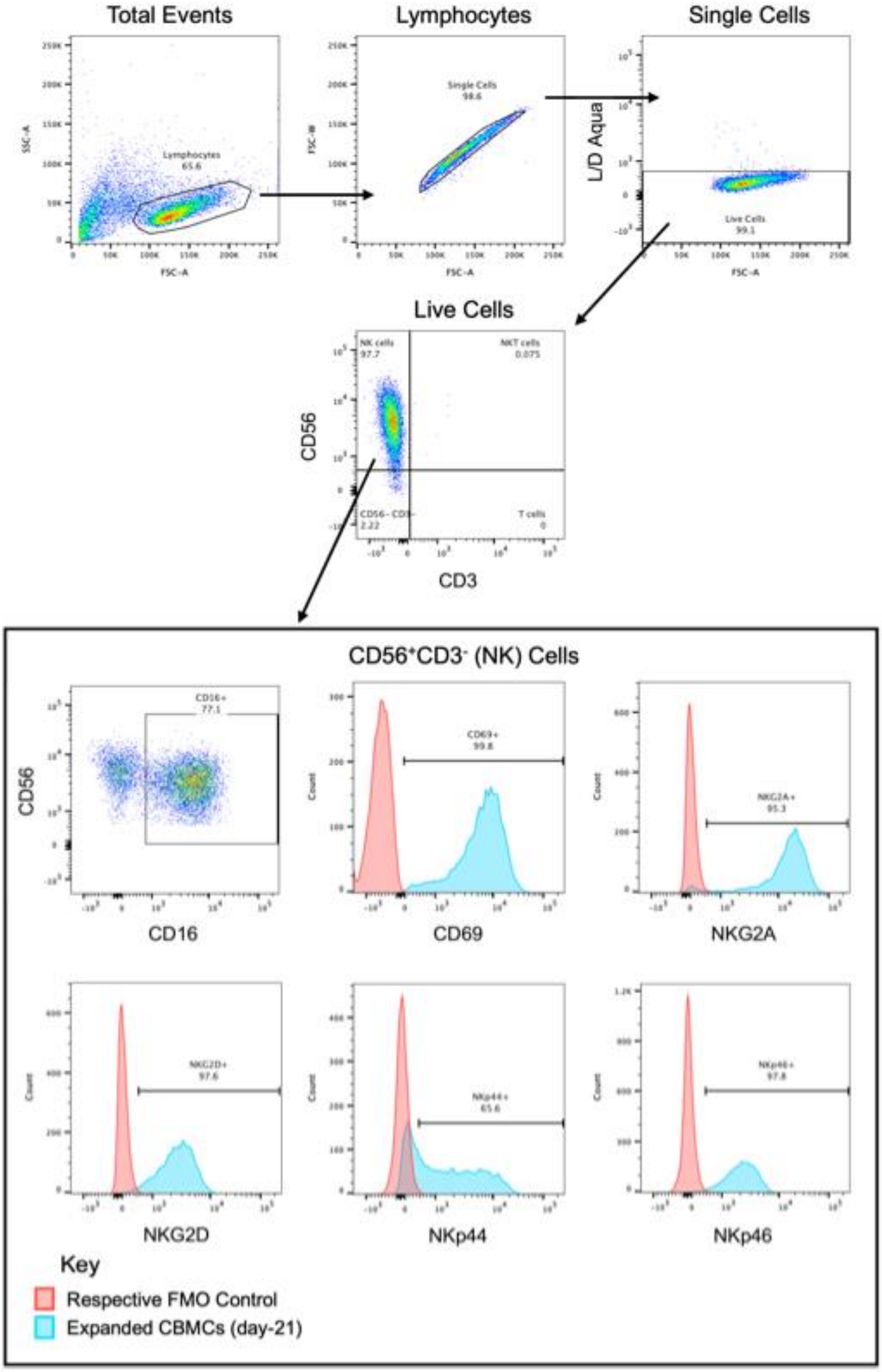

Supplementary Table 1. Antibody panels used for flow cytometry. Cocktails 1 and 2 were used for phenotypic characterisation of UCB and APB derived NK cells. Cocktail 3 was used for the flow-based cytoxicity assay, (a) for calculating the percentage of target cell lysis, and (b) for measurement of intracellular IFN-y and TNF-a expression. Cocktail 4 was used to conduct enhanced phenotyping of expanded UCB-derived NK cells post-cryopreservation.

Cocktail	Antibody/Stain	Cat No.	Manufacturer
1	CD3-FITC	555339	BD
	CD56-APC	555518	BD
	CD16-Alexafluor 700	557920	BD
	CD69-PE-CF594	562617	BD
	NKG2D-PE	563688	BD
	NKp46-PE-Cy7	562101	BD
	LIVE/DEAD Fixable Aqua Dead Cell Stain Kit	L34966	Invitrogen
2	CD3-FITC	555339	BD
	CD56-APC	555518	BD
	NKG2A-PE	130-113-566	Miltenyi
	NKp44-PE-Cy7	325116	Biolegend
	LIVE/DEAD Fixable Aqua Dead Cell Stain Kit	L34966	Invitrogen
3	CD3-FITC	555339	BD
	CD56-APC	555518	BD
	CD107a-PE-CF594	562628	BD
	Zombie NIR Fixable Viability Kit	423106	Biolegend
3a	IFNy-BV421	502532	Biolegend
	TNFa-PE	130-120-489	Miltenyi
4	CD3-FITC	555339	BD
	CD56-APC	555518	BD
	CD16-Alexafluor 700	557920	BD
	CD69-PE-CF594	562617	BD
	NKG2A-PE	130-113-566	Miltenyi
	NKG2D-BV711	563688	BD
	NKp44-PE-Cy7	325116	Biolegend
	NKp46-BV421	564065	BD
	CD2-PE-Cy5	300210	Biolegend
	NKp30-BV605	325234	Biolegend
	CD158b/j-APC-Fire750	312618	Biolegend
	CD158b2-PerCP-Vio700	130-126-093	Miltenyi
	Zombie NIR Fixable Viability Kit	423106	Biolegend

Supplementary Figure 3. Purity and Identity of CD3 depleted CBMCs. *Graph showing the percentage of residual CD3+ cells present in the population following isolation (CBMC isolation and CD3 depletion), on the day of thawing and after 7, 14 and 21 days in culture.*

- *(A) For cells isolated using magnetic beads and MACS columns (Miltenyi Biotech)*
- *(B) For cells isolated using FACS (Tyto- Miltenyi Biotech) and*
- *(C) Phenotypic Identity of cells following CD3 depletion (MACS columns) the mean of $CD3^{-}CD56^{+}$ cells is 51.7±15.2%)*

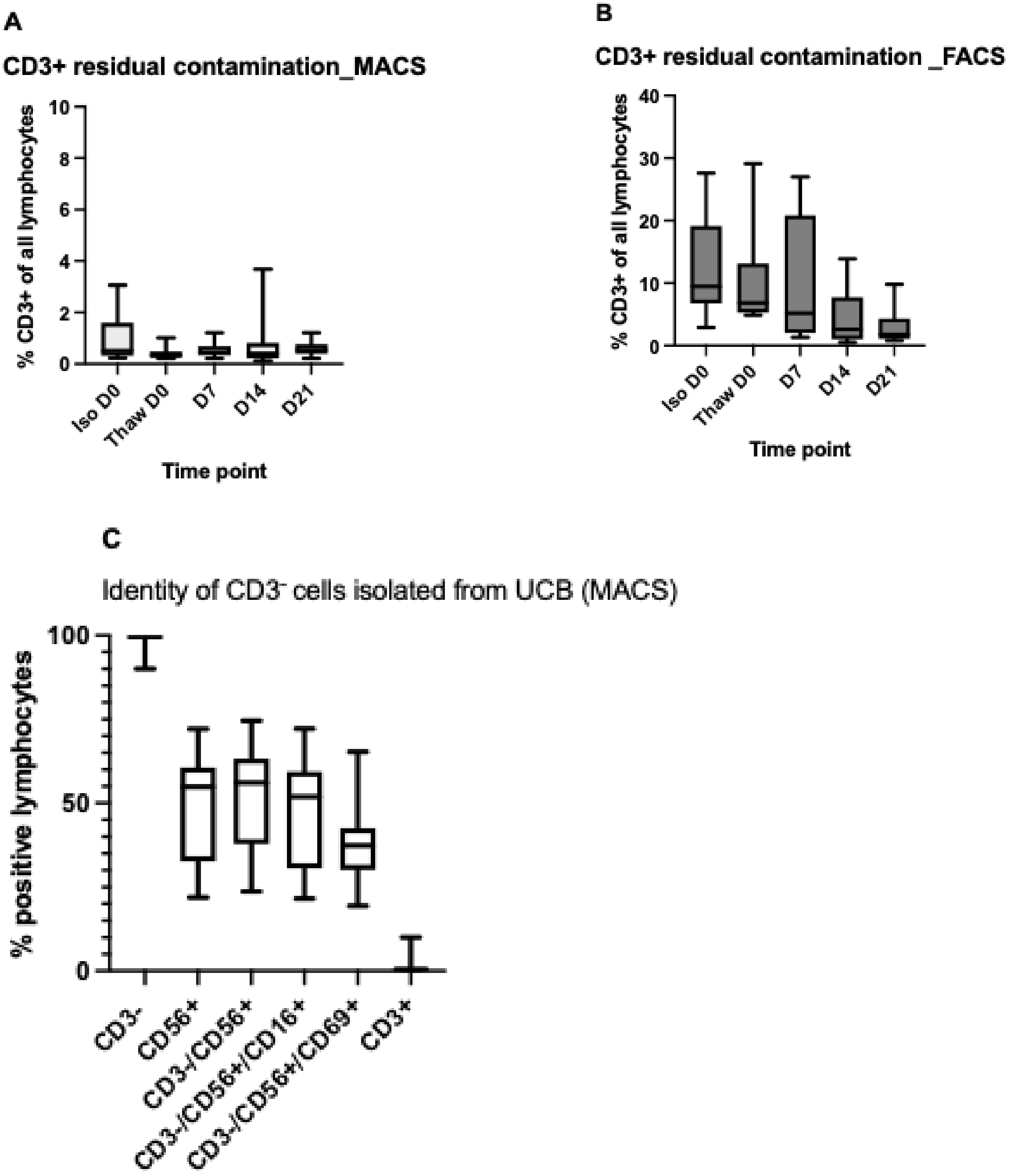

Supplementary table 2. Mean and standard deviation values used for the correlational matrix.

	Cohort			
	Fresh Starting Cells		Cryopreserved Starting Cells	
	**Mean**	**Std. Deviation**	**Mean**	**Std. Deviation**
D21 Fold Expansion	62.19	± 35.37	283.8	± 126.5
D21 Cytotoxicity	44.72	± 11.53	49.04	± 12.11
Gestational Age (d)	278.1	± 7.955	281.5	± 6.983
Birthweight (g)	3439	± 473.5	3737	± 600.5
Maternal Age (y)	32.59	± 7.33	32.23	± 5.464
No. White Blood Cells (WBC; E+03/uL)	9.025	± 2.434	10.73	± 2.663
No. Red Blood Cells (RBC; E+06/uL)	2.95	± 0.4763	2.925	± 0.2637
Red Cell Distribution Width (RDW-SD; fL)	73.62	± 6.812	73.37	± 7.542
Platlet Density Width (PDW; fL)	10.04	± 1.426	9.992	± 1.146
Lymphocyte (% of WBC diff)	35.33	± 9.434	33.79	± 6.484
%Monocyte	10.6	± 2.383	11.73	± 2.705
No. Nucleated Red Blood Cells (NRBC; E+03/uL)	0.6286	± 0.71	0.48	± 0.3847
Total Nuclear Count (TNC)	98.02	± 36.29	116.2	± 29.99
No. Mononuclear Cells excluding NRBCs (E+07)	40.65	± 14.23	49.66	± 11.33
Sample Weight (g)	133.2	± 18.86	143	± 10.72
Sample Volume (ml)	99.04	± 16.52	104	± 10.16
Time to Refridgeration (m)	28.34	± 15.71	31.08	± 8.281
Time to Isolation (h)	43.79	± 7.735	19.38	± 3.341

Supplementary Figure 4. Correlational matrix heatmaps with full annotation of p values and r values. (A) Corresponds to figure 3A in the manuscript. (B) Corresponds to figure 5A in the manuscript.

A

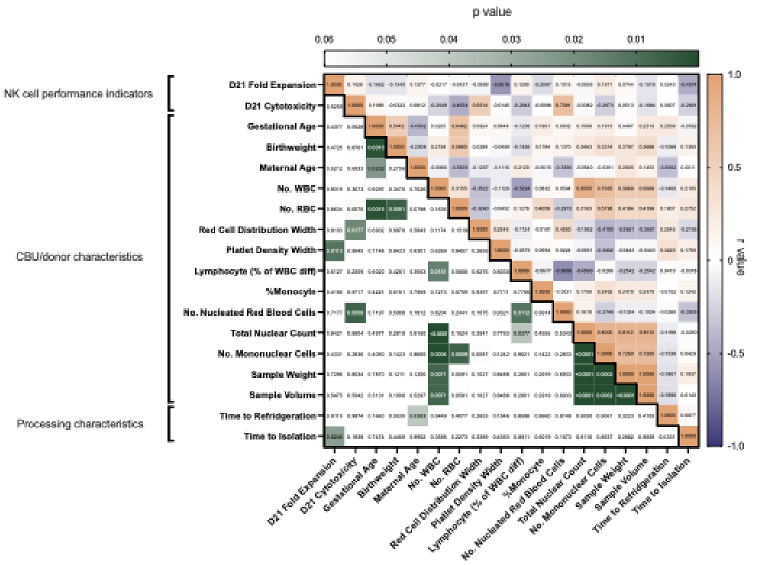

B

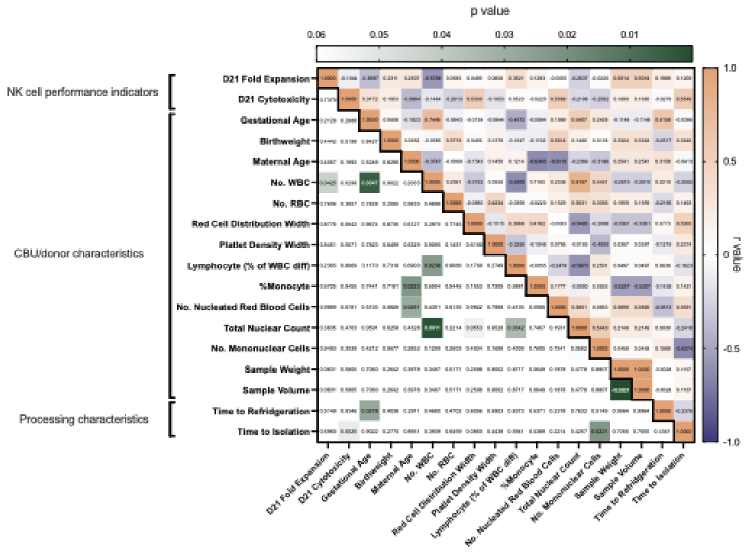

Supplementary Figure 5. The subdivision of the delivery types between the vaginal deliveries (VD) and caesarean sections (CS) in the two UCB sample groups for this study plotted against day 21 NK cell fold expansion.

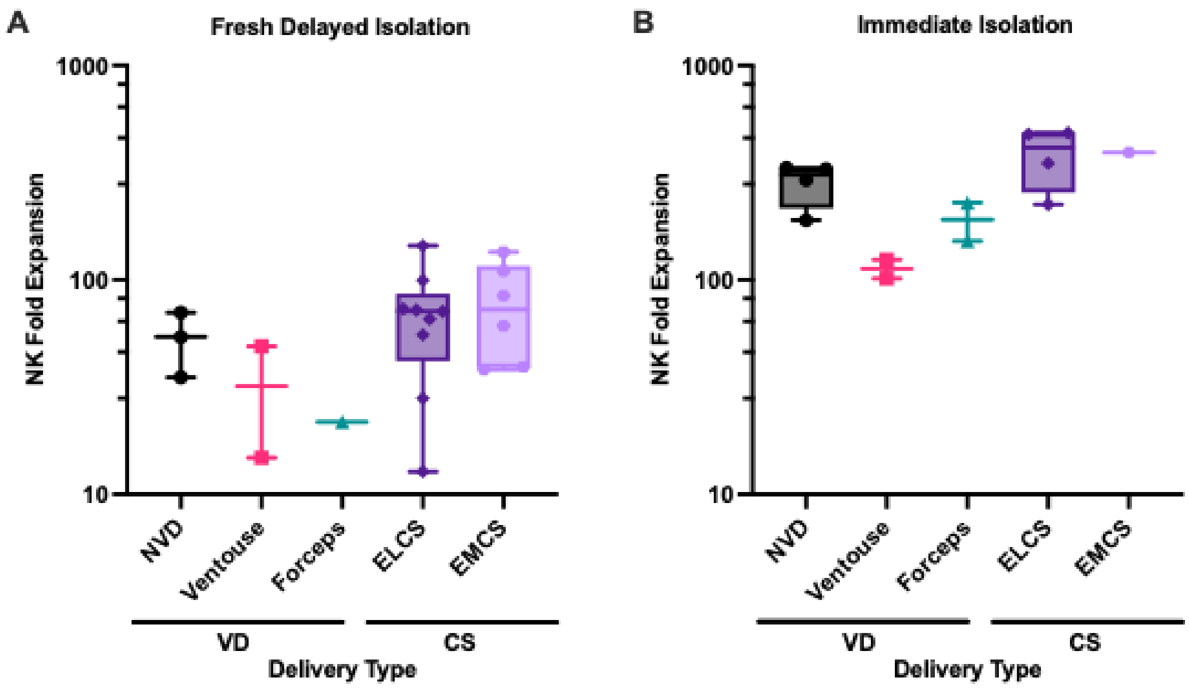

Supplementary Fig 6. Equation used to calculate the approximate predicted yield of NK cells per 1 x 10^8 CBMCs used for $CD3^-$ CBMC isolation and NK cell expansion in a feeder free system.

Approximate predicted yield was calculated for each CBU that was collected from a CS delivery and processed in the optimal way (NK cells expanded from cryopreserved $CD3^-$ CBMCs that were isolated immediately; n=5). Using the number of live $CD3^-$ CBMCs recovered post-thaw per 1 x 10^8 CBMCs input into the $CD3^+$ cell depletion, the initial percentage of NK cells and the NK specific fold expansion. The predicted yield was calculated from expansion data from each CBU then the average was taken (1.14 x 10^9 ± 3.398 x 10^8).

$$\text{Predicted Yield} = \frac{\text{initial }\%\text{CD56}^{+}\text{CD3}^{-} \times \text{no. live cells post thaw}}{100} \times \text{NK cell fold expansion}$$

Supplementary Fig 7. Flow cytometry plots (forward and side scatter) showing the leukocyte populations on UCB derived cells after (A) initial isolation, (B) following freeze/thaw and after (C) 7 days and (D) 14 days in culture. The differences in the proportions of the leukocyte populations, suggest a preferential loss of monocytes over time which starts after the freeze/thaw cycle, and continues through the culture period (the same cord sample was used for each of the plots)

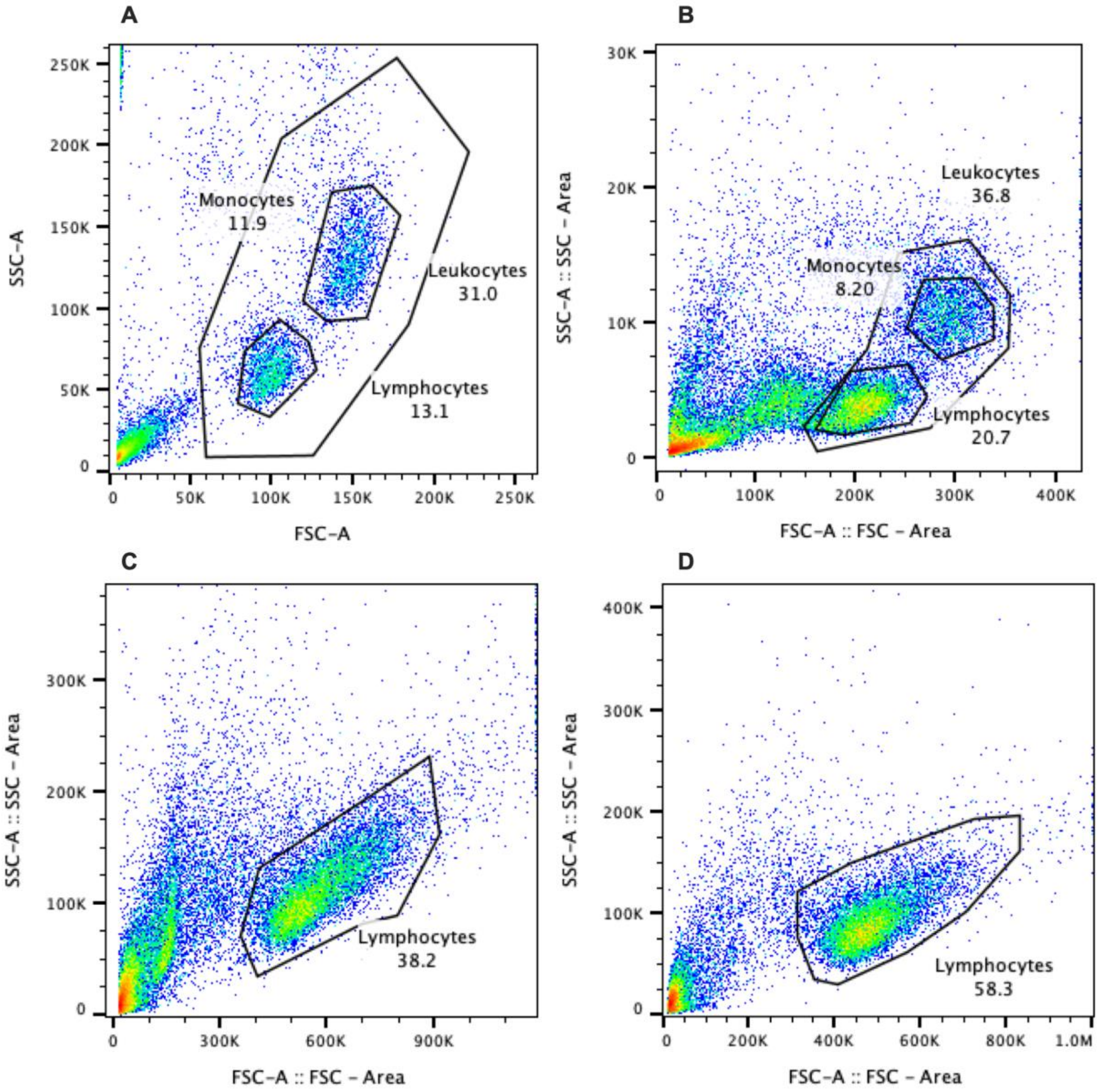

Supplement: Supplementary file 1 [file DataSheet1.pdf]
